# The US Public’s Perception of the Threat of COVID-19 During the Rapid Spread of the COVID-19 Outbreak: Cross-Sectional Survey Study

**DOI:** 10.2196/23400

**Published:** 2021-02-08

**Authors:** Xiaolei Xiu, Anran Wang, Qing Qian, Sizhu Wu

**Affiliations:** 1 Department of Medical Data Sharing Institute of Medical Information & Library Chinese Academy of Medical Sciences & Peking Union Medical College Beijing China

**Keywords:** COVID-19, perceived threat of disease, environment, behavior, America

## Abstract

**Background:**

The rapid spread of the COVID-19 pandemic in the United States has made people uncertain about their perceptions of the threat of COVID-19 and COVID-19 response measures. To mount an effective response to this epidemic, it is necessary to understand the public's perceptions, behaviors, and attitudes.

**Objective:**

We aimed to test the hypothesis that people’s perceptions of the threat of COVID-19 influence their attitudes and behaviors.

**Methods:**

This study used an open dataset of web-based questionnaires about COVID-19. The questionnaires were provided by Nexoid United Kingdom. We selected the results of a questionnaire on COVID-19–related behaviors, attitudes, and perceptions among the US public. The questionnaire was conducted from March 29 to April 20, 2020. A total of 24,547 people who lived in the United States took part in the survey.

**Results:**

In this study, the average self-assessed probability of contracting COVID-19 was 33.2%, and 49.9% (12,244/24,547) of the respondents thought that their chances of contracting COVID-19 were less than 30%. The self-assessed probability of contracting COVID-19 among women was 1.35 times that of males. A 5% increase in perceived infection risk was significantly associated with being 1.02 times (OR 1.02, 95% CI 1.02-1.02; *P*<.001) more likely to report having close contact with >10 people, and being 1.01 times (OR 1.01, 95% CI 1.01-1.01; *P*<.001) more likely to report that cohabitants disagreed with taking steps to reduce the risk of contracting COVID-19. However, there was no significant association between participants who lived with more than 5 cohabitants or less than 5 cohabitants (*P*=.85). Generally, participants who lived in states with 1001-10,000 COVID-19 cases, were aged 20-40 years, were obese, smoked, drank alcohol, never used drugs, and had no underlying medical conditions were more likely to be in close contact with >10 people. Most participants (21,017/24,547, 85.6%) agreed with washing their hands and maintaining social distancing, but only 20.2% (4958/24,547) of participants often wore masks. Additionally, male participants and participants aged <20 years typically disagreed with washing their hands, maintaining social distancing, and wearing masks.

**Conclusions:**

This survey is the first attempt to describe the determinants of the US public’s perception of the threat of COVID-19 on a large scale. The self-assessed probability of contracting COVID-19 differed significantly based on the respondents’ genders, states of residence, ages, body mass indices, smoking habits, alcohol consumption habits, drug use habits, underlying medical conditions, environments, and behaviors. These findings can be used as references by public health policy makers and health care workers who want to identify populations that need to be educated on COVID-19 prevention and health.

## Introduction

### Background

COVID-19 is an acute infectious respiratory disease that is caused by the novel SARS-CoV-2 virus [1]. It was first detected on December 2019 in Wuhan, China. SARS-CoV-2 is a novel coronavirus that has not been previously identified in humans. It also has a stronger ability to spread and a longer incubation period than the severe acute respiratory syndrome coronavirus. Therefore, SARS-CoV-2 poses a greater epidemic risk [2]. In January 29, 2020, 7711 COVID-19 cases were confirmed in China, and 98 cases were confirmed in 18 other countries [3,4]. As a result, the World Health Organization (WHO) declared the COVID-19 epidemic a public health emergency of international concern on January 30, 2020, and called on all countries to collaborate and prevent the spread of the COVID-19 epidemic [5]. At the beginning of the outbreak, the Chinese government immediately initiated rigorous and intensive prevention and control measures to contain the spread of SARS-CoV-2. Such measures included implementing effective medical treatments, initiating precautionary measures, conducting comprehensive testing, and reducing social mobility and social contact by implementing travel restrictions, banning large gatherings, closing public places, and issuing a stay-at-home order [6,7]. However, due to the strong human-to-human transmissibility of the SARS-CoV-2 virus, the COVID-19 epidemic spread rapidly around the world. On March 11, 2020, the WHO declared the COVID-19 epidemic a worldwide pandemic.

The United States announced the first confirmed case of COVID-19 on January 21, 2020, and the SARS-CoV-2 virus spread to all 50 states by mid-March. As of April 28, 2020, the cumulative number of confirmed cases in the United States has exceeded 1,000,000 [8]. To control the development of the COVID-19 epidemic, the US government implemented a number of measures, including enhancing detection capacities, increasing investments in medical resources, limiting social activities, and providing economic assistance. However, the number of confirmed cases is still rapidly growing at a rate of more than 20,000 new cases per day. The continuous development of the COVID-19 epidemic has seriously affected the physical and mental health of the US public. In terms of coping with large-scale epidemics, the effects of policy implementation are usually influenced by group behaviors. People’s methods for coping with large-scale epidemics depend on each individual's perception of the risk of contracting the disease and each individual’s ability to adjust their behavior when adapting to environmental changes, to a certain extent [9]. To ensure ultimate success in the fight against COVID-19, it is necessary for the public to adhere to the control measures that have been proposed by the government. However, compliance with control measures depends on the perceived threat level of COVID-19. Therefore, to control the pandemic and quickly reduce the impact of the epidemic on the US economy and society, we must understand the US public's perception of the threat of COVID-19. This will provide the health sector with useful information on increasing disaster preparedness and improving countermeasures.

### Risk Perception During the COVID-19 Epidemic

Risk perceptions refer to people’s intuitive evaluations of hazards that they are or might be exposed to [10]. Risk perception is influenced by multiple individual and societal factors, such as different social, cultural, and contextual factors. Risk perceptions act as triggers for precautionary action [11]. When individuals and communities deem risks as unsafe, unacceptable, or something to be feared, their responses and adherence to important public health measures for risk mitigation will be influenced [12]. For example, risk perceptions may shape the public’s willingness to accept and adhere to COVID-19 risk mitigation strategies, such as social distancing and the use of face masks [13]. As the number of COVID-19–related deaths rises around the world, understanding the public’s risk perceptions becomes increasingly more important [14].

Researchers around the world have been actively conducting COVID-19 surveys. Research on the perception of COVID-19 risk mainly focuses on the following 3 aspects: (1) the influencing factors of risk perception (eg, personality characteristics, knowledge level, and health and economy status) and their relationship with protective behaviors [15-17]; (2) the risk perceptions of COVID-19 among special populations, such as college students under quarantine, medical students, patients with COVID-19, health care professionals, and pregnant women [18-22]; and (3) the impact of risk perception on social distancing and the mental health of the public or health workers (eg, anxiety and distress) [23-27]. However, there are few studies on the American public’s risk perception of COVID-19. For instance, Wise et al [28] investigated risk perceptions and self-reported protective behaviors among 1591 individuals who lived in the United States during the first week of the outbreak. Furthermore, Niepel et al [29] discussed the risk perception of COVID-19–related fatality among adult US residents. However, there is limited scientific evidence for personality characteristics, risk perceptions, attitudes, and behavior patterns among the American public, especially with regard to the attitudes and behaviors of cohabitants. In addition, most research on the United States is based on adults; there is a lack of research on the risk perceptions of COVID-19 among American people aged <18 years. Moreover, the sample sizes of existing surveys have been limited (ie, generally hundreds or thousands of samples), which has led to certain limitations with regard to the interpretation of the results. Additionally, there have been no COVID-19 surveys that target American people of all ages and have a very large sample size (ie, tens of thousands of samples).

This paper describes a cross-sectional web-based survey that was designed to gauge the US public’s perception of the threat posed by COVID-19. This study is one of the first attempts to conduct a large-scale survey for determining the US public’s perception of the health threat of COVID-19, and the characteristics that influence the US public’s environment and behaviors toward COVID-19.

## Methods

### Participants

The dataset for this cross-sectional study came from the COVID-19 (Coronavirus) Survival Calculator project [30]. This project was created and managed by the data research team at Nexoid United Kingdom, which is a software company that is located in London, United Kingdom and specializes in research, analysis, and computer science. The COVID-19 (Coronavirus) Survival Calculator is a website that was posted on Reddit (ie, a discussion-based platform) under the subreddit r/Coronavirus_PH. The website was launched to measure participants’ risk rates of SARS-CoV-2 infection [30]. The website is available in 49 languages, such as English, Spanish, Portuguese, Russian, and French, and it asks participants certain questions in a survey-like manner (ie, participants can choose the following 3 answers: disagree, neutral, or agree). The dataset is protected by the Creative Commons Attribution 4.0 International (CC BY 4.0) License. The dataset is available for everyone to download, but its respondents remain anonymous for privacy and safety reasons.

On April 20, 2020, Nexoid United Kingdom released the original data of 682,793 surveys. Data were collected from March 24 to April 20, 2020. This open dataset contains the longitude and latitude data of participants' Internet Protocol (IP) addresses. To find respondents who participated in the survey in the United States, we used the Baidu Map geocoder to parse and convert latitude and longitude data, so that we could obtain information on participants’ locations. However, to protect participants’ privacy, Nexoid United Kingdom does not publish participants’ IP addresses. As such, our IP address location data were accurate up to 5 km. Therefore, there may have been several errors in participants’ location information in this study.

The dataset initially included 566,122 respondents from the United States. However, we excluded participants who had missing values in the “chance of getting COVID-19” field and participants whose gender was labelled as “other.” Furthermore, we conducted stratified random sampling so that the distribution of participants in the age and gender subgroups matched that of the general population, as per the US Census Bureau’s methodology when they reported demographic data for the 2019 US census [31]. The respondents participated in this cross-sectional study from March 29 to April 20, 2020. This study was performed 10 weeks after the first COVID-19 case was confirmed in the United States. This survey period corresponds to the rapid spread of the COVID-19 outbreak in the United States.

### Data Availability

The data supporting this study is openly available in the Population Health Data Archive [32].

### Measures

The COVID-19 (Coronavirus) Survival Calculator is rich in content, such as demographic characteristics, environmental factors, behavioral factors, governmental factors, and health-related factors. In addition, the calculator includes 2 questions about people’s perceptions of the threat posed by COVID-19. These questions assessed a respondent’s chance of contracting COVID-19 and a respondent’s chance of dying from COVID-19. Before participants accessed the survey, Nexoid United Kingdom provided a data and privacy statement, which informed participants that the calculator would record the data that they enter on the page and their locations (ie, locations derived from participants’ IP addresses). In addition, participants were informed that the survey was anonymous, the collected data would be added to a freely accessible dataset, and the dataset would be shared.

For the purposes of this study, we selected specific data from the COVID-19 (Coronavirus) Survival Calculator, such as demographics, environmental factors, behavioral factors, and the people’s chances of contracting COVID-19. Demographic characteristics included gender, the place of current residence, age, body mass index (BMI), smoking status, alcohol consumption status, nonprescription/recreational drug use (ie, cannabis, amphetamines, cocaine, lysergic acid diethylamide, and 3,4-methylenedioxymethamphetamine use), and underlying medical conditions. Several questions had too many answer options in the original questionnaire, which is not conducive to the analysis of practical problems. Therefore, we regrouped the answer options for several questions.

As per the classification criteria of the Centers for Disease Control and Prevention (CDC), participants’ areas of residence were divided into the following six categories, based on the number of confirmed COVID-19 cases that were reported by participants’ state of residence: 0-1000 cases, 1001-5000 cases, 5001-10,000 cases, 10,001-20,000 cases, 20,001-40,000 cases, and ≥40,001 cases. Participants were also divided into the following four age categories: 0-20 years, 20-40 years, 40-60 years, and >60 years. Furthermore, participants were divided into the following weight categories based on the WHO BMI criteria: underweight (BMI<18.5 kg/m^2^), normal weight (BMI=18.5-24.9 kg/m^2^), preobesity (BMI=25-29.9 kg/m^2^), and obesity (BMI≥30 kg/m^2^). In addition, smoking status was divided into four categories (ie, never, quit, vape, and yes), and alcohol consumption status (ie, never, none in last 14 days, and some in last 14 days) and drug use status (ie, never, none in last 28 days, and some in last 28 days) were divided into three categories. Underlying medical conditions included asthma, carcinoma, chronic kidney disease, compromised immune system, coronary heart disease, chronic obstructive lung disease, diabetes, HIV disease, hypertension, or other chronic illness. We categorized participants’ underlying medical condition status as either “have” or “none.”

The questionnaire in this study contained seven questions (Table 1). Of these 7 questions, 3 pertained to environmental factors, 3 asked about behavioral factors, and 1 addressed people’s perceptions of the threat posed by COVID-19. The question that addressed people’s perceptions of the threat posed by COVID-19 provided participants with the following 10 answer options: 0%-10%, 10%-20%, 20%-30%, 30%-40%, 40%-50%, 50%-60%, 60%-70%, 70%-80%, 80%-90%, and 90%-100%. The questionnaire used the median of each probability range as participants’ final results (eg, 5%, 15%, and 25%).

**Table 1:** Questionnaire on the environmental and behavioral factors that affect people’s perceptions of the risk of contracting COVID-19.

Topic, question, and options			Value	
**Environment**				
	**How many people were you in close contact with in the last week, including partners, children, work colleagues, customers, patients, etc? (number of close contacts), mean (SD)**			6.9 (6.6)
		<10 people, n (%)	19,772 (80.5)	
		>10 people, n (%)	4774 (19.5)	
	**How many people live in your house/apartment? (number of cohabitants), mean (SD)**			3.1 (1.6)
		<5 people, n (%)	22,741 (92.6)	
		>5 people, n (%)	1806 (7.4)	
	**Do you travel to work/school?**			
		Home (I always worked/studied from home), n (%)	1439 (5.9)	
		Never (I did not go to work/school before), n (%)	5003 (20.4)	
		Stopped (I have stopped going to work/school), n (%)	10,469 (42.6)	
		Critical travel (I still go to work; critical job fields include health care, utilities, military, etc), n (%)	5703 (23.2)	
		Noncritical travel (ie, I still go to work/school), n (%)	1933 (7.9)	
**Behaviors**				
	**I am taking steps to reduce my risk (eg, social distancing, washing hands, etc)**			
		Disagree, n (%)	397 (1.6)	
		Neutral, n (%)	3133 (12.8)	
		Agree, n (%)	21,017 (85.6)	
	**The people I live with are taking steps to reduce my risk (social distancing, washing hands)**			
		Disagree, n (%)	740 (3)	
		Neutral, n (%)	4956 (20.2)	
		Agree, n (%)	18,851 (76.8)	
	**Do you wear a mask when outside of your house/flat?**			
		Rarely, n (%)	14,053 (57.2)	
		Sometimes, n (%)	4981 (20.3)	
		Usually, n (%)	5513 (22.5)	
**Opinion of infection (% chance), mean (SD)**			33.2 (21.8)	
	**What do you think are your** **chances of getting COVID-19?**			
		0%-10%, n (%)	4236 (17.3)	
		10%-20%, n (%)	3865 (15.7)	
		20%-30%, n (%)	4143 (16.9)	
		30%-40%, n (%)	3061 (12.5)	
		40%-50%, n (%)	3434 (14)	
		50%-60%, n (%)	3110 (12.7)	
		60%-70%, n (%)	1195 (5.9)	
		70%-80%, n (%)	868 (3.5)	
		80%-90%, n (%)	355 (1.4)	
		90%-100%, n (%)	280 (1.1)	

### Statistical Analysis

We described the frequency of participants’ demographic characteristics and various environmental and behavioral factors. Independent samples 2-tailed *t* tests, one-way analysis of variance tests, or Chi-square tests were used to compare the differences between groups of categorical variables, as appropriate. Furthermore, we used Bonferroni-corrected *P* values for multiple comparisons. In our logistic regression analysis, all demographic, environmental, and behavioral variables were used as independent variables, and participants’ self-assessments of their chances of contracting COVID-19 were used as the outcome variable. The logistic regression analysis was conducted to identify factors that were associated with participants’ perceptions of the threat of contracting COVID-19. We conducted binary logistic regression analyses to identify factors that were associated with environmental factors and behaviors. Factors were selected by using the backward stepwise method. Odds ratios and their 95% confidence intervals were used to quantify the associations among variables, estimated probabilities of infection, environmental factors, and behaviors. Results were considered statistically significant when *P*<.05. All analyses were conducted from May 2020 to June 2020 with SPSS version 23.0 (IBM Corp).

## Results

After excluding 97,883 respondents who had missing values in the “chance of getting COVID-19” field, 1742 respondents whose gender was labelled as “other,” and 441,950 respondents due to stratified random sampling, the final sample consisted of 24,547 participants. These respondents lived in all 50 states in the United States. Of the 24,547 respondents in the final sample, 12,465 (50.8%) were women, 6100 (24.9%) were aged <20 years, 6677 (27.2%) were aged 20-40 years, 6191 (25.2%) were aged 40-60 years, and 5579 (22.7%) were aged >60 years. Furthermore, the average BMI of the respondents was 29.41 kg/m^2^ (SD 7.74 kg/m^2^; range 11.6-87.6 kg/m^2^), and 39.2% (9617/24,547) of the respondents were obese (BMI≥30 kg/m^2^). Additionally, 56.7% (13,919/24,547) of the participants reported that they never smoked, 51.5% (12,632/24,547) reported that they drank alcohol in the last 14 days before taking the survey, and 47.3% (11,621/24,547) reported that they never used drugs. In terms of medical conditions, 61% (14,983/24,547) of participants did not have the underlying medical conditions that were described in this study. Additional demographic information is included in Table 2.

**Table 2:** Participants’ demographic characteristics and self-assessment results for their risk of contracting COVID-19, stratified by demographic variables (N=24,547).

Demographic characteristics		Number of participants, n (%)	Opinion of infection (% chance), mean (SD)	*t* test (df) or *F* test (df)	*P* value
**Gender**				10.99^a^ (24,464.28)	<.001
	Female	12465 (50.8)	34.70 (21.44)		
	Male	12082 (49.2)	31.65 (22.01)		
**Number of COVID-19 cases in a participant’s state of current residence**				5.83^b^ (5)	<.001
	0-1000	606 (2.5)	33.07 (22.39)		
	1001-5000	5326 (21.7)	33.91 (21.87)		
	5001-10,000	3638 (14.8)	32.71 (21.46)		
	10,001-20,000	6937 (23.3)	32.96 (21.71)		
	20,001-40,000	6834 (27.8)	32.71 (21.63)		
	≥40,001	1206 (4.9)	35.80 (22.97)		
**Age (years)**				163.72^b^ (3)	<.001
	0-20	6100 (24.9)	31.33 (21.00)		
	20-40	6677 (27.2)	36.59 (22.05)		
	40-60	6191 (25.2)	35.28 (22.16)		
	>60	5579 (22.7)	28.89 (20.92)		
**BMI^c^**				18.68^b^ (3)	<.001
	Underweight (BMI<18.5 kg/m^2^)	568 (2.3)	31.55 (21.13)		
	Normal weight (BMI=18.5-24.9 kg/m^2^)	7136 (29.1)	32.15 (21.61)		
	Preobesity (BMI=25-29.9 kg/m^2^)	7226 (29.4)	32.70 (21.64)		
	Obesity (BMI≥30 kg/m^2^)	9617 (39.2)	34.46 (21.98)		
**Smoking status**				10.52^b^ (3)	<.001
	Never	13919 (56.7)	32.61 (21.53)		
	Quit	5374 (21.9)	33.42 (21.92)		
	Vape	2189 (8.9)	34.21 (21.46)		
	Yes	3065 (12.5)	34.78 (22.72)		
**Alcohol consumption status**				48.22^b^ (2)	<.001
	Never	6165 (25.1)	30.95 (21.70)		
	None in last 14 days	5750 (23.4)	33.27 (21.87)		
	Some in last 14 days	12632 (51.5)	34.27 (21.69)		
**Nonprescription/recreational drug use^d^**				52.52^b^ (2)	<.001
	Never	11621 (47.3)	31.86 (21.74)		
	None in last 28 days	6491 (26.4)	34.15 (21.67)		
	Some in last 28 days	5065 (20.6)	35.33 (21.68)		
**Underlying medical conditions**				−10.93^a^ (24,545)	<.001
	None	14983 (61)	31.99 (21.60)		
	Have	9564 (39.0)	35.10 (21.92)		

^a^A 2-tailed *t* test value.

^b^An *F* test value.

^c^BMI: body mass index; participants had a mean BMI of 29.41 kg/m^2^ (SD 7.74 kg/m^2^).

^d^The total number of participants in this category does not equal 24,547 due to missing data.

In terms of environmental factors, the average number of people that the respondents had close contact with in the week before taking the survey was 6.9 people (SD 6.6 people) (Table 1). In addition, 19.5% (4774/24,547) of participants reported that they had close contact with more than 10 people. In our sample, an average of 3.1 people (SD 1.6 people) lived together in 1 house/apartment, and 92.6% (22,741/24,547) of the respondents reported that less than five people lived in their houses/apartments. Furthermore, 42.6% (10,469/24,547) of the participants stopped working and going to school, while 7.9% (1933/24,547) were still in school or engaged in noncritical work.

In terms of behaviors, most participants (21,017/24,547, 85.6%) were willing to take measures to reduce the risk of infection, but 1.6% (397/24,547) of participants reported that they were not willing to take risk reduction measures. Additionally, 76.8% (18,851/24,547) of the respondents stated that their cohabitants were also taking steps to reduce the risk of infection. However, 57.2% (14,053/24,547) of the respondents reported that they rarely or never wore a mask, and only 22.5% (5513/24,547) stated that they often wore masks.

The average self-assessed probability of COVID-19 infection was 33.2% (SD 21.8%; range 5%-95%); 33% (8101/24,547) of the respondents thought their chances of contracting COVID-19 were <20%, but 2.5% (635/24,547) thought they had a >80% chance of contracting COVID-19 (Table 1). Moreover, participants’ perceptions of the threat posed by COVID-19 significantly differed across genders (*P*<.001), places of residence (*P*<.001), ages (*P*<.001), BMI categories (*P*<.001), smoking statuses (*P*<.001), alcohol consumption statuses (*P*<.001), nonprescription/recreational drug use statuses (*P*<.001), and underlying medical condition statuses (*P*<.001) (Table 2).

The logistic regression analysis results suggested that there were several important relationships between variables (Table 3). Women thought that their chances of contracting COVID-19 was 1.35 times (OR 1.35, 95% CI 1.28-1.41) that of males. The self-assessed probabilities of contracting COVID-19 for participants who lived in states with 5001-10,000, 10,001-20,000, and 20,001-40,000 COVID-19 cases were 22% (OR 0.78, 95% CI 0.70-0.88; OR 0.78, 95% CI 0.70-0.87; OR 0.78, 95% CI 0.70-0.87, respectively) lower than those of participants who lived in states with >40,000 cases. The self-assessed probabilities of contracting COVID-19 for participants aged 0-20 years, 20-40 years, and 40-60 years were 1.32 times (OR 1.32, 95% CI 1.22-1.43), 1.68 times (OR 1.68, 95% CI 1.56-1.81) and 1.55 times (OR 1.55, 95% CI 1.44-1.66) higher than those of participants aged >60 years, respectively. The self-assessed infection probabilities of participants with a normal BMI were 6% (OR 0.94, 95% CI 0.88-0.99) lower than those of participants who were obese. The self-assessed probabilities of contracting COVID-19 for participants who did not smoke or quit smoking were 8% (OR 1.08, 95% CI 1.00-1.16; OR 1.08, 95% CI 1.00-1.18) higher than those of participants who smoked cigarettes. The self-assessed probabilities of participants who did not drink and did not take drugs were 13% (OR 0.87, 95% CI 0.82-0.92) and 23% (OR 0.77, 95% CI 0.72-0.82) lower than those of participants who did drink and take drugs, respectively. Moreover, participants without underlying medical conditions were 32% (OR 0.68, 95% CI 0.65, 0.72) less likely to contract COVID-19 than participants with these conditions, as per their self-assessment results. Furthermore, compared to participants who had close contact with more than 10 people, participants who were in close contact with less than 10 people had a 43% (OR 0.57, 95% CI 0.53, 0.61) lower self-assessed probability of contracting COVID-19. Participants who engaged in critical work assessed that their probability of contracting COVID-19 was 1.50 times (OR 1.50, 95% CI 1.37-1.65) higher than those of participants who engaged in noncritical work. In addition, participants who were neutral about taking steps to reduce their infection risk thought that they were 21% (OR 0.79, 95% CI 0.73, 0.85) less likely to contract COVID-19 compared to those who agreed with taking steps to reduce their infection risk.

The self-assessed probability of contracting COVID-19 for participants who lived with people that did not take steps to reduce their infection risk were 1.56 times (OR 1.56, 95% CI 1.36-1.79) higher than those of participants who lived with people that did take measures for reducing their infection risk. Respondents who rarely wore masks assessed that their probability of contracting COVID-19 was 34% (OR 0.76, 95% CI 0.72-0.81) lower than that of participants who frequently wore masks.

**Table 3:** Results of the logistic regression analysis of factors that were associated with participants’ self-assessed probability of contracting COVID-19.

Categories			B (SE)	OR (95% CI)	*P* value^a^
**Demographic characteristics**					
	**Gender**				
		Female	.30 (.02)	1.35 (1.28-1.41)^a^	<.001
		Male	0^b^	1.0 (referent)	N/A^c^
	**Number of COVID-19 cases in a participant’s state of current residence**				
		0-1000	−.23 (.09)	0.79 (0.67-0.95)	.01
		1001-5000	−.16 (.06)	0.85 (0.76-0.95)	.005
		5001-10,000	−.24 (.06)	0.78 (0.70-0.88)	<.001
		10,001-20,000	−.25 (.06)	0.78 (0.70-0.87)	<.001
		20,001-40,000	−.25 (.06)	0.78 (0.70-0.87)	<.001
		≥40,001	0^b^	1.0 (referent)	N/A
	**Age (years)**				
		0-20	.28 (.04)	1.32 (1.22-1.43)	<.001
		20-40	.52 (.04)	1.68 (1.56-1.81)	<.001
		40-60	.44 (.04)	1.55 (1.44-1.66)	<.001
		>60	0^b^	1.0 (referent)	N/A
	**BMI^d^**				
		Underweight (BMI<18.5 kg/m^2^)	−.03 (.08)	0.97 (0.83-1.13)	.67
		Normal weight (BMI=18.5-24.9 kg/m^2^)	−.07 (.03)	0.94 (0.88-0.99)	.02
		Preobesity (BMI=25-29.9 kg/m^2^)	−.04 (.03)	0.96 (0.91-1.01)	.13
		Obesity (BMI≥30 kg/m^2^)	0^b^	1.0 (referent)	N/A
	**Smoking status**				
		never	.07 (.04)	1.08 (1.00-1.16)	.053
		Quit	.08 (.04)	1.08 (1.00-1.18)	.05
		Vape	.08 (.05)	1.09 (0.98-1.20)	.11
		Yes	0^b^	1.0 (referent)	N/A
	**Alcohol consumption status**				
		Never	−.14 (.03)	0.87 (0.82-0.92)	<.001
		None in last 14 days	−.12 (.03)	0.89 (0.84-0.94)	<.001
		Some in last 14 days	0^b^	1.0 (referent)	N/A
	**Nonprescription/recreational drugs use status**				
		Never	−.26 (.03)	0.77 (0.72-0.82	<.001
		None in last 28 days	−.11 (.03)	0.90 (0.84-0.96)	.002
		Some in last 28 days	0^b^	1.0 (referent)	N/A
	**Underlying medical conditions**				
		None	−.38 (.03)	0.68 (0.65-0.72)	<.001
		Have	0^b^	1.0 (referent)	N/A
**Environment**					
	**Number of close contacts**				
		<10 people	−.57 (.04)	0.57 (0.53-0.61)	<.001
		<10 people	0^b^	1.0 (referent)	N/A
	**Number of cohabitants**				
		<5 people	.06 (.04)	1.06 (0.97-1.16)	.17
		> 5 people	0^a^	1.0 (referent)	N/A
	**Going to work/school**				
		Home	−.12 (.06)	0.89 (0.78-1.01)	.06
		Never	.30 (.05)	0.74 (0.67-0.82)	<.001
		Stopped	−.04 (.05)	0.96 (0.87-1.05)	.33
		Critical travel	.41 (.05)	1.50 (1.37-1.65)	<.001
		Noncritical travel	0^b^	1.0 (referent)	N/A
**Behaviors**					
	**Taking steps to reduce infection risk**				
		Disagree	−.52 (.10)	0.60 (0.49-0.72)	<.001
		Neutral	−.24 (.04)	0.79 (0.73-0.85)	<.001
		Agree	0^b^	1.0 (referent)	N/A
	**Cohabitants are taking steps to reduce infection risk**				
		Disagree	.45 (.07)	1.56 (1.36-1.79)	<.001
		Neutral	.15 (.03)	1.17 (1.09-1.24)	<.001
		Agree	0^b^	1.0 (referent)	N/A
	**Wearing a mask**				
		Rarely	−.27 (.03)	0.76 (0.72-0.81)	<.001
		Sometimes	.01 (.04)	1.01 (0.94-1.09)	.72
		Usually	0^b^	1.0 (referent)	N/A

^a^Bonferroni *P* values are used to correct for multiple comparisons. Statistical significance was set to a level of *P*<.05.

^b^These parameters were set to 0 because they were redundant.

^c^N/A: not appliable.

^d^BMI: body mass index.

The binary logistic regression analysis results revealed several predictors among the environmental factors (Table 4). When participants’ self-assessed risks of contracting COVID-19 increased by 5%, they were 1.02 times (OR 1.02, 95% CI 1.02-1.02) more likely to report that they had close contact with more than 10 people, while their odds of reporting a cessation of work/school decreased by 1% (OR 0.99, 95% CI 0.99-0.99). The odds of having close contact with more than 10 people increased by 82% (OR 1.82, 95% CI 1.52-2.18), 89% (OR 1.89, 95% CI 1.57-2.28), 58% (OR 1.58, 95% CI 1.32-1.89), and 45% (OR 1.45, 95% CI 1.21-1.73) for participants who lived in states with 1001-5000, 5001-10,000, 10,001-20,000, and 20,001-40,000 COVID-19 cases, respectively, compared to those who lived in states with >40,001 confirmed COVID-19 cases. The odds of reporting a cessation of work/school decreased by 34% (OR 0.66, 95% CI 0.58-0.75), 32% (OR 0.68, 95% CI 0.59-0.78), 22% (OR 0.78, 95% CI 0.68-0.88), and 26% (OR 0.86, 95% CI 0.74-0.95) for participants who lived in states with 1000-5000, 5001-10,000 cases, 10,001-20,000, and 20,001-40,000 COVID-19 cases, respectively, compared to those who lived in states with >40,001 COVID-19 cases. Compared to participants aged 20-40 years, participants aged 40-60 years were 30% less likely (OR 0.70, 95% CI 0.64-0.76) to have close contact with more than 10 people in the week before taking the survey, and 14% less likely (OR 0.86, 95% CI 0.75-0.99) to live with more than five people. Participants aged <20 years were 1.68 times (OR 1.68, 95% CI 1.56-1.81) more likely to report that they ceased to go to work/school compared to those aged 20-40 years. Participants who were obese were 1.28 times more likely (OR 1.28, 95% CI 1.18-1.39) to have close contact with more than 10 people compared to participants with a normal BMI. Participants who smoked electronic cigarettes and cigarettes were also 1.41 times (OR 1.41, 95% CI 1.26-1.58) and 1.68 times (OR 1.68, 95% CI 1.52-1.86) more likely to have close contact with more than 10 people compared to nonsmokers, respectively. Participants who drank alcohol within 14 days before taking the survey were 1.30 times more likely (OR 1.30, 95% CI 1.19-1.42) to have close contact with more than 10 people compared to participants who never drank alcohol. However, participants who were obese were 24% less likely (OR 0.76, 95% CI 0.71-0.81) to stop going to work/school compared to participants with a normal BMI, and participants who smoked cigarettes were 42% less likely (OR 0.58, 95% CI 0.53-0.63) to report that they stopped going to work/school compared to nonsmokers. Compared to participants who never used drugs, participants who used drugs in the last 28 days before taking the survey were 21% less likely (OR 0.79, 95% CI 0.72-0.87) to have close contact with more than 10 people. Participants who never used drugs were also 18% more likely (OR 1.18, 95% CI 1.09-1.27) to cease going to school/work compared to participants who did use drugs. Participants who had underlying medical conditions were 16% less likely (OR 0.84, 95% CI 0.79-0.91) to have close contact with more than 10 people and 9% more likely (OR 1.09, 95% CI 1.03-1.15) to stop going to school/work compared to participants who did not have underlying medical conditions.

**Table 4:** Results of the binary logistic regression analysis of factors that were significantly associated with environmental factors.

Demographic characteristics			Close contact with >10 people			Living with >5 cohabitants				Stopped going to work/school		
			OR (95% CI)	*P* value		OR (95% CI)		*P* value		OR (95% CI)		*P* value
Opinion of infection			1.02 (1.02-1.02)^a^	<.001		1.00 (1.00-1.00)		.85		0.99 (0.99-0.99)^a^		<.001
**Gender**												
	Female	0.96 (0.90-1.02)		.21	1.10 (1.00-1.22)		.053		1.11 (1.05-1.17)^a^		<.001	
	Male	1.0 (referent)		N/A^b^	1.0 (referent)		N/A		1.0 (referent)		N/A	
**Number of COVID-19 cases in a participant’s state of current residence**												
	0-1000	1.25 (0.94-1.66)		.12	1.13 (0.77-1.66)		.53		0.80 (0.66-0.98)^c^		.03	
	1001-5000	1.82 (1.52-2.18)^a^		<.001	1.08 (0.84-1.40)		.54		0.66 (0.58-0.75)^a^		<.001	
	5001-10,000	1.89 (1.57-2.28)^a^		<.001	1.09 (0.84-1.42)		.54		0.68 (0.59-0.78)^a^		<.001	
	10,001-20,000	1.58 (1.32-1.89)^a^		<.001	1.12 (0.88-1.44)		.36		0.78 (0.68-0.88)^a^		<.001	
	20,001-40,000	1.45 (1.21-1.73)^a^		<.001	1.07 (0.83-1.37)		.61		0.84 (0.74-0.95)^d^		.005	
	≥40,001	1.0 (referent)		N/A	1.0 (referent)		N/A		1.0 (referent)		N/A	
**Age (years)**												
	0-20	1.05 (0.96-1.15)		.34	2.05 (1.80-2.33)^a^		<.001		1.68 (1.56-1.81)^a^		<.001	
	20-40	1.0 (referent)		N/A	1.0 (referent)		N/A		1.0 (referent)		N/A	
	40-60	0.70 (0.64-0.76)^a^		<.001	0.86 (0.75-0.99)^d^		.04		1.03 (0.96-1.10)		.48	
	>60	0.32 (0.28-0.36)^a^		<.001	0.25 (0.20-0.31)^a^		<.001		0.50 (0.46-0.55)^a^		<.001	
**BMI^e^**												
	Underweight (BMI<18.5 kg/m^2^)	0.82 (0.65, 1.03)		.09	0.87 (0.65-1.16)		.35		1.13 (0.94-1.35)		.19	
	Normal weight (BMI=18.5-24.9 kg/m^2^)	1.0 (referent)		N/A	1.0 (referent)		N/A		1.0 (referent)		N/A	
	Preobesity (BMI=25-29.9 kg/m^2^)	1.16 (1.06-1.27)^d^		.001	1.01 (0.88-1.15)		.91		0.88 (0.82-0.94)^a^		<.001	
	Obesity (BMI≥30 kg/m^2^)	1.28 (1.18-1.39)^a^		<.001	1.06 (0.94, 1.20)		.32		0.76 (0.71-0.81)^a^		<.001	
**Smoking status**												
	Never	1.0 (referent)		N/A	1.0 (referent)		N/A		1.0 (referent)		N/A	
	Quit	1.06 (0.97-1.17)		.19	1.02 (0.88-1.18)		.80		0.83 (0.62-0.89)^a^		<.001	
	Vape	1.41 (1.26-1.58)_a_		<.001	1.13 (0.96-1.33)		.15		0.68 (0.73-0.75)^a^		<.001	
	Yes	1.68 (1.52-1.86)^a^		<.001	1.37 (1.17-1.60)^a^		<.001		0.58 (0.53-0.63)^a^		<.001	
**Alcohol consumption status**												
	Never	1.0 (referent)		N/A	1.0 (referent)		N/A		1.0 (referent)		N/A	
	None in last 14 days	1.38 (1.25-1.53)^a^		<.001	0.94 (0.82-1.08)		.39		0.95 (0.88-1.03)		.24	
	Some in last 14 days	1.30 (1.19-1.42)^a^		<.001	0.72 (0.64-0.82)^a^		<.001		1.17 (1.10-1.26)^a^		<.001	
**Nonprescription/recreational drug use status**												
	Never	1.0 (referent)		N/A	1.0 (referent)		N/A		1.0 (referent)		N/A	
	None in last 28 days	0.87 (0.80-0.94)^c^		.001	0.91 (0.80-1.03)		.14		1.16 (1.09-1.24)^a^		<.001	
	Some in last 28 days	0.79 (0.72-0.87)^a^		<.001	0.90 (0.78-1.04)		.15		1.18 (1.09-1.27)^a^		<.001	
**Underlying medical conditions**												
	Have	0.84 (0.79-0.91)^a^		<.001	1.05 (0.95-1.17)		.37		1.09 (1.03-1.15)^d^		.003	
	None	1.0 (referent)		N/A	1.0 (referent)		N/A		1.0 (referent)		N/A	

^a^Significant at a level of *P*<.001.

^b^N/A: not applicable.

^c^Significant at a level of *P*<.05.

^d^Significant at a level of *P*<.01.

^e^BMI: body mass index.

The binary logistic regression analysis results revealed several predictors among behaviors (Table 5). When participants’ self-assessed risks of contracting COVID-19 increased by 5%, they were 1.01 times (OR 1.01, 95% CI 1.01-1.01) more likely to report that their cohabitants disagreed with taking steps to reduce the risk of contracting COVID-19, and their odds of reporting that they rarely wore masks decreased by 1% (OR 0.99, 95% CI 0.99-0.99). The odds of women disagreeing with taking measures to reduce their infection risk and rarely wearing masks were 40% (OR 0.60, 95% CI 0.49-2.74) and 25% (OR 0.75, 95% CI 0.71-0.79) lower than those of males, respectively. Compared to those who lived in states with >40,001 confirmed COVID-19 cases, participants from states with 1001-5000 cases and 5001-10,000 cases were 1.98 times (OR 1.98, 95% CI 1.73-2.25) and 1.80 times (OR=1.80, 95% CI: 1.57-2.06) more likely to report that they rarely wore masks, respectively. Compared to participants aged 20-40 years, participants aged <20 years were 2.96 times (OR 2.96, 95% CI 2.27-3.88), 2.04 times (OR 2.04, 95% CI 1.68-2.47) and 1.48 times (OR 1.48, 95% CI 1.36-1.60) more likely to report that they did not take protective measures, their cohabitants did not take protective measures, and they rarely wore masks, respectively. Participants who were obese were 1.14 times (OR 1.14, 95% CI 1.06-1.21) more likely to report that they rarely wore masks compared to participants with a normal BMI. Compared to nonsmokers, participants who smoked cigarettes were 2.22 times (OR 2.22, 95% CI 1.65-2.99), 1.57 times (OR 1.57, 95% CI 1.25-1.96) and 1.11 times (OR 1.11, 95% CI 1.02-1.21) more likely to report that they did not take protective measures, their cohabitants did not take protective measures, and they rarely wore masks, respectively. Participants who drank alcohol were 1.18 times (OR 1.18, 95% CI 1.10-1.27) more likely to report that they rarely wore masks compared to participants who never drank alcohol. Participants who used drugs within 28 days before answering the survey were 1.47 times (OR 1.47, 95% CI 1.20-1.81) more likely to report that their cohabitants did not agree with taking measures to reduce infection risk compared to participants who never used drugs. Additionally, participants who had underlying medical conditions were 1.31 times (OR 1.31, 95% CI 1.12-1.53) more likely to report that their cohabitants did not agree with measures to reduce infection risk compared to participants without these conditions.

**Table 5:** Results of the binary logistic regression analysis of significant behavioral factors.

Demographic characteristics		Disagree with taking steps to reduce the risk of contracting COVID-19				Cohabitants disagree with taking steps to reduce the risk of contracting COVID-19				Rarely wears a mask			
		OR (95% CI)		*P* value		OR (95% CI)		*P* value		OR (95% CI)		*P* value	
Opinion of infection		1.00 (0.99-1.00)		.22		1.01 (1.01-1.01)^a^		<.001		0.99 (0.99-1.00)^a^		<.001	
**Gender**													
	Female		0.60 (0.49-0.74)^a^		<.001		1.30 (1.12-1.51)^c^		.001		0.75 (0.71-0.79)^a^		<.001
	Male		1.0 (Referent)		N/A^b^		1.0 (Referent)		N/A		1.0 (Referent)		N/A
**Number of COVID-19 cases in a participant’s state of current residence**													
	0-1000		1.76 (0.83-3.75)		.14		1.20 (0.71-2.05)		.495		1.59 (1.30-1.95)^a^		<.001
	1001-5000		1.32 (0.76-2.29)		.33		0.95 (0.66-1.36)		.76		1.98 (1.73-2.25)^a^		<.001
	5001-10,000		1.34 (0.76-2.37)		.32		0.82 (0.56-1.20)		.30		1.80 (1.57-2.06)^a^		<.001
	10,001-20,000		1.15 (0.66-1.99)		.62		0.99 (0.69-1.41)		.95		1.62 (1.43-1.84)^a^		<.001
	20,001-40,000		1.19 (0.69-2.06)		.53		0.87 (0.61-1.24)		.43		1.31 (1.15-1.49)^a^		<.001
	≥40,001		1.0 (Referent)		N/A		1.0 (Referent)		N/A		1.0 (Referent)		N/A
**Age (years)**													
	0-20		2.96 (2.27-3.88)^a^		<.001		2.04 (1.68-2.47)^a^		<.001		1.48 (1.36-1.60)^a^		<.001
	20-40		1.0 (Referent)		N/A		1.0 (Referent)		N/A		1.0 (Referent)		N/A
	40 to 60 years		0.52 (0.35-0.76)^c^		.001		0.46 (0.35-0.59)^a^		<.001		0.68 (0.63-0.73)^a^		<.001
	More than 60 years		0.69 (0.47-1.01)		.06		0.56 (0.43-0.72)^a^		<.001		0.42 (0.39-0.46)^a^		<.001
**BMI^d^**													
	Underweight (BMI<18.5 kg/m^2^)		1.10 (0.68-1.76)		.70		1.17 (0.81-1.67)		.41		1.07 (0.89-1.29)		.47
	Normal weight (BMI=18.5-24.9 kg/m^2^)		1.0 (Referent)		N/A		1.0 (Referent)		N/A		1.0 (Referent)		N/A
	Preobesity (BMI=25-29.9 kg/m^2^)		0.71 (0.54-0.93)^e^		.014		0.73 (0.59-0.89)^c^		.002		1.06 (0.99-1.13)		.12
	Obesity (BMI≥30 kg/m^2^)		1.03 (0.81-1.31)		.81		0.88 (0.73-1.04)		.13		1.14 (1.06-1.21)^a^		<.001
**Smoking status**													
	Never		1.0 (Referent)		N/A		1.0 (Referent)		N/A		1.0 (Referent)		N/A
	Quit		1.31 (0.96-1.78)		.09		1.18 (0.95-1.46)		.14		0.97 (0.91-1.04)		.42
	Vape		1.69 (1.25-2.30)^c^		.001		0.94 (0.73-1.22)		.65		1.03 (0.93-1.14)		.54
	Yes		2.22 (1.65-2.99)^a^		<.001		1.57 (1.25-1.96)^a^		<.001		1.11 (1.02-1.21)^c^		.019
**Alcohol consumption status**													
	Never		1.0 (Referent)		N/A		1.0 (Referent)		N/A		1.0 (Referent)		N/A
	None in last 14days		1.12 (0.83-1.52)		.47		0.98 (0.80-1.21)		.88		1.17 (1.08-1.27)^a^		<.001
	Some in last 14 days		1.31 (0.99-1.73)		.056		0.82 (0.67-1.00)^e^		.047		1.18 (1.10-1.27)^a^		<.001
**Nonprescription/recreational drug use status**													
	Never		1.0 (Referent)		N/A		1.0 (Referent)		N/A		1.0 (Referent)		N/A
	None in last 28 days		1.03 (0.78-1.36)		.87		1.15 (0.94, 1.41)		.16		0.98 (0.91-1.04)		.46
	Some in last 28 days		1.30 (0.99-1.71)		.06		1.47 (1.20-1.81)^a^		<.001		0.82 (0.76-0.89)^a^		<.001
**Underlying medical conditions**													
	Have		0.89 (0.71-1.12)		.32		1.31 (1.12-1.53)^c^		.001		0.84 (0.80-0.89)^a^		<.001
	None		1.0 (Referent)		N/A		1.0 (Referent)		N/A		1.0 (Referent)		N/A

^a^Significant at a level of *P*<.001.

^b^N/A: not appliable.

^c^ Significant at a level of *P*<.01.

^d^BMI: body mass index.

^e^Significant at a level of *P*<.05.

## Discussion

**Principal Results**

This study analyzed the US public’s perception of the threat posed by COVID-19 during the rapid spread of the COVID-19 pandemic. Individuals’ risk perceptions affect their protective behaviors during the early stages of a pandemic [28,33]. By extending this idea to the COVID-19 epidemic, we hypothesized that when people believe that they have an increased chance of contracting COVID-19, they will be more willing to follow public health recommendations. This study showed that a high self-assessed probability of contracting COVID-19 was related to the following factors: having close contact with more than 10 people, working at a critical job, having cohabitants that disagreed with taking steps to reduce infection risk, and sometimes wearing masks outside the house. In addition, this study also analyzed environmental factors, behaviors, and their associations with people’s self-assessed probabilities of contracting COVID-19. We also identified several demographic factors that were associated with environmental factors, behaviors, and people’s self-assessed probabilities of contracting COVID-19. These findings can be used as references by public health policy makers and health care workers who want to identify populations that need to be educated on COVID-19 prevention and health education.

The sample’s average self-assessed probability of contracting COVID-19 was 33.2%, and 49.9% (12,399/24.547) of the participants believed that their chances of contracting COVID-19 were less than 30%. This indicated that most respondents were optimistic about the COVID-19 pandemic. Moreover, participants’ self-assessed probabilities of contracting COVID-19 differed significantly across respondents’ genders, participants’ states of residence, ages, BMIs, smoking statuses, alcohol consumption statuses, drug use statuses, underlying disease statuses, environments, and COVID-19–related behaviors. Participants who were obese, were aged 20-40 years, consumed alcohol in the last 14 days before taking the survey, took drugs in the last 28 days before taking the survey, and had underlying medical conditions thought that they had a higher chance of contracting COVID-19. These findings are in line with those of other literature about controversial scientific topics [34-36].

In this study, women felt that they had a higher chance of contracting COVID-19 than men. Women’s risk perceptions of COVID-19 also affect their protective behaviors. Women pay more attention to self-protection methods, such as agreeing with taking measures to reduce the risk of contracting COVID-19 and wearing masks, compared to men. However, Chen et al [37] found that more men contracted COVID-19 than women. Studies have also shown that the mortality rate of men is higher than that of women [38]. Nonetheless, in this study, male participants were more reluctant to take measures for reducing their infection risk and less willing to wear masks compared to female participants. These environmental factors and behaviors greatly increase the chance of contracting COVID-19.

We also found that young people (ie, aged <20 years) were more reluctant to wear masks and take measures for reducing the risk of contracting COVID-19 compared to older people. Although the vast majority of COVID-19–related deaths occur in older patients and those with underlying health conditions, this does not mean that young people cannot be infected with SARS-CoV-2. Studies have shown that anyone can become severely ill from contracting COVID-19. The SARS-CoV-2 virus has been infecting young people, and this has resulted in young people spreading COVID-19 [39].

In this study, cigarette smokers reported that their chances of contracting COVID-19 were lower than those of nonsmokers. Although there are no peer-reviewed studies that have evaluated the association between the risk of SARS-CoV-2 infection and smoking, available research suggests that smokers are at a high risk of developing severe COVID-19 outcomes and dying [40,41]. Therefore, it may be necessary to strengthen COVID-19–related prevention and health education for men, young people, and smokers, to increase their understanding of COVID-19.

In general, participants who lived in states where the epidemic was more severe thought that they had a high chance of contracting COVID-19. Therefore, they also paid more attention to maintaining social distance and taking protective measures. These participants were more likely to stop going to work/school and often wear masks. It is worth noting that although the outbreak was less severe in states with 1001-5000 confirmed COVID-19 cases, participants in these states paid less attention to maintaining social distance, were the least likely to stop going to school/work, and were the least willing to take protective measures and wear masks. In addition, these participants thought they were less likely to contract COVID-19 than those who lived in states with more than 40,001 cases. Environmental factors and people’s behaviors toward COVID-19 are likely to result in an increased number of confirmed cases in states with 1001-5000 COVID-19 cases. According to the data provided by USA Facts [8], in April 20, 2020, the states with 1001-5000 reported cases were Arkansas, Delaware, Idaho, Iowa, Kansas, Kentucky, Minnesota, Mississippi, Nebraska, Nevada, New Hampshire, New Mexico, Oklahoma, Oregon, South Carolina, South Dakota, Utah, and Wisconsin. A week later, the number of confirmed cases in Arkansas, Delaware, Iowa, Kansas, and Nebraska increased by more than 50%, and the number of cases in Nebraska increased by 122.74%. In addition, the number of COVID-19 cases in Iowa, Mississippi, South Carolina, and Wisconsin exceeded 5000. Therefore, to avoid a resurgence of COVID-19, people who live in low-risk areas cannot ignore daily preventive measures.

Limiting face-to-face contact with others is the best way to reduce the spread of COVID-19. During the COVID-19 outbreak (ie, March 29 to April 20, 2020) 42.6% (10,469/24,547) of participants stopped going to work/school. This was related to the closure of many schools and businesses in the United States on March 2, 2020 [8]. However, 19.5% (4774/24,547) of the participants reported that they had close contact with more than 10 people in the week before taking the survey. This went against the White House’s recommendation on March 16, 2020 (ie, this survey was conducted between March 29 and April 20, 2020) [42]. Those who lived in states with 5001-10,000 cases, were aged 20-40 years, were obese, and smoked had a greater chance of reporting that they had close contact with more than 10 people. In the 2 weeks after the lockdown in Hubei Province, China, only 3.6% of people reported that they went to crowded places [43]. It is possible that China's stringent isolation measures and the Chinese government’s strong enforcement of these measures motivated Chinese citizens to comply with government orders. Unlike in China, there is still some debate between the federal US government and the state governments about the need to implement stay-at-home orders and similar measures. Stay-at-home orders are predominantly issued by state governments rather than the federal government. On March 19, 2020, California was the first state to issue a stay-at-home order. As of March 29, 2020, 26 states, such as Illinois, New Jersey, New York, and Washington, have issued similar orders [44]. More than half of Americans are required to stay at home to maintain social distance and reduce close contact with each other. Moreover, between March 29 and April 20, 2020, an additional 17 states and Washington, D.C. issued stay-at-home orders. This may have led the population to change their behaviors toward crowds.

Health care workers are among the most vulnerable groups because of their proximity to patients with COVID-19. In our sample, participants who had critical jobs (eg, health care jobs, utilities jobs, military jobs, etc) also thought they had a high probability of contracting COVID-19 (mean 40.81%, SD 23.05%; Multimedia Appendix 1). In spite of this, only 20.2% (1151/5703) of participants with critical jobs reported that they wore masks regularly, and 57.3% (3267/5703) reported that they rarely wore masks. The lack of protective equipment has further increased the risk of infection in people with critical jobs. According to a report from The Hill [45], on March 19, 2020, health care workers were forced to reuse single-use masks and protective equipment due to shortages caused by the COVID-19 epidemic. The US Department of Health and Human Services have reported that there was a serious shortage of new COVID-19 tests for medical staff and a shortage of protective equipment on a large scale. During the COVID-19 pandemic, the strong risk of infection and the shortage of protective equipment have caused great stress, anxiety, fear and other strong emotions in people with critical jobs. Studies have shown that hospital medical staff who are in charge of patients with COVID-19 have a higher incidence of mental symptoms, such as somatization, obsessive-compulsive behavior, anxiety, hostility, and paranoia, than the general public. Furthermore, hospital medical staff have been experiencing obvious psychological, behavioral, and emotional problems [46,47]. Therefore, in addition to increasing the supply of protective equipment, psychological interventions and counseling should be provided to people with critical jobs, to address their psychological needs.

In this study, participants had different attitudes toward social distancing, washing hands, and wearing a mask. We found that 85.6% (21,017/24,547) of participants agreed with taking steps to reduce the risk of contracting COVID-19 (eg, social distancing and washing hands), while only 22.5% (5513/24,547) usually wore masks. In China, only 2% of people have reported that they do not wear masks outside of home [43]. This is not only related to the personality of US citizens, but also to the decisions of the US government. In China, people have been encouraged—and even mandated—to wear masks outside of home. The CDC has encouraged people to wash their hands frequently and avoid close contact with people. However, the CDC has also recommended that healthy people should not use masks, to ensure that masks are available for frontline health care workers [48]. In Western countries, people do not need to wear a mask unless they are sick. The use of masks is still an evolving process. Many studies and practices have shown that wearing masks is important for slowing the spread of SARS-CoV-2 [49-51]. On April 3, 2020, the CDC revised its guidelines for wearing masks, and recommended that people should wear cloth face coverings in public settings where other social distancing measures are difficult to maintain. Since then, the proportion of US residents who wear masks has rapidly increased.

**Limitations**

This study has several limitations. First, the scope of our investigation was limited. The recruitment of participants was based on their willingness to participate and access to social networking sites. As such, our participants are not representative of the entire American public. Furthermore, participants were not compensated for participating in this survey. This may reflect participants’ prior concerns about contracting COVID-19. Therefore, the respondents may have a higher self-assessment risk of contracting COVID-19 compared to that of the general population. However, we conducted stratified random sampling to achieve a distribution of participants that matched the general population in terms of age and sex, thereby reducing selection bias to a certain extent. Second, people’s perceptions of the threat posed by COVID-19 are influenced by many factors, and these factors are constantly changing. The questionnaire in this study asked participants to answer very specific questions that could not comprehensively cover the complex situations surrounding participants’ awareness of their chances of getting COVID-19. For example, the content of the Nexoid United Kingdom questionnaire was updated on April 25, 2020; Nexoid United Kingdom added factors such as private health insurance, income, and race. However, these factors are not included in this study.

**Conclusions**

In summary, this survey is the first large-scale attempt to describe the determinants of the US public's perception of the threat posed by COVID-19. We also describe COVID-19–related environmental factors and behaviors in the United States, and their relationship with the public’s perceptions. Our findings suggest that the US public is generally optimistic; they believe that they have a relatively low chance of contracting COVID-19 during the rapid spread of the COVID-19 outbreak. People, especially women, who live in high-risk areas, are obese, smoke, drink, take drugs, and have chronic or immune-related diseases think that they have a high risk of contracting COVID-19. These people are also more anxious. The struggle to control the spread of COVID-19 will be long and drawn out. Considering the likely future resurgence of COVID-19, it is important to consider implementing specific policies and programs for those who are severely affected by the pandemic, to control the epidemic as soon as possible. Due to the limited questionnaire content, it is necessary to conduct more research on other factors (eg, income and race) that affect the US public's assessment of their chances of contracting COVID-19.

## Appendix

Multimedia Appendix 1 Supplemental materials.

## Abbreviations

BMI: body mass index
CDC: Centers for Disease Control and Prevention
IP: Internet Protocol
WHO: World Health Organization
