# Supplementary material for: The US Public’s Perception of the Threat of COVID-19 During the Rapid Spread of the COVID-19 Outbreak: Cross-Sectional Survey Study
Source: J Med Internet Res. 2021 Feb 8;23(2):e23400. doi: 10.2196/23400 (PMC7871980; doi:10.2196/23400)
Supplement: Multimedia Appendix 1 [file jmir_v23i2e23400_app1.docx]

**The US Public’s Perception of the Threat of COVID-19 During the Rapid Spread of the COVID-19 Outbreak: Cross-sectional Survey Study**

We here provide additional figures and analyses.

### Survey date


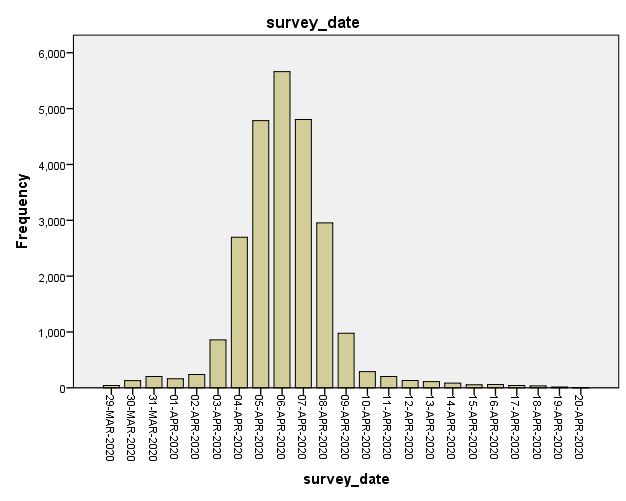


Figure S1. Questionnaire survey time and number of participants per day

### Follow-up analysis for Table 3 in the main document

Comparison of demographic variables, environmental variables and behavioral variables (post-hoc tests)

#### *Independent Samples Test——Gender*

|  |  | N | Mean | Std. Deviation | Std. Error Mean |
| --- | --- | --- | --- | --- | --- |
| Opinion_infection | female | 12465 | 34.702 | 21.4413 | .1920 |
|  | male | 12082 | 31.654 | 22.0117 | .2003 |

|  | | F | Sig. | t | df | Sig.  (2-tailed) | Mean Difference |
| --- | --- | --- | --- | --- | --- | --- | --- |
|  |  |  |  |  |  |  |  |
| Opinion_infection | Equal variances assumed | 25.200 | .000 | 10.989 | 24545 | .000 | 3.0479 |
|  | Equal variances not assumed |  |  | 10.985 | 24464.277 | .000 | 3.0479 |

#### *ANOVA - Place of current residence*

|  | Sum of Squares | df | Mean Square | F | Sig. |
| --- | --- | --- | --- | --- | --- |
| Between Groups | 13809.301 | 5 | 2761.860 | 5.830 | .000 |
| Within Groups | 11626682.931 | 24541 | 473.766 |  |  |
| Total | 11640492.231 | 24546 |  |  |  |

**Post Hoc Comparisons - Place of current residence**

| (I) Place_of_current_residence | (J) Place_of_current_residence | Mean Difference (I-J) | Std. Error | Sig. |
| --- | --- | --- | --- | --- |
| 40001 or more cases | 0 to1000 cases | 2.7350 | 1.0838 | .174 |
|  | 1001 to 5000 cases | 1.8933 | .6941 | .096 |
|  | 5001 to 10000 cases | 3.0940^*^ | .7232 | .000 |
|  | 10001 to 20000 cases | 2.8455^*^ | .6791 | .000 |
|  | 20001 to 40000 cases | 3.0958^*^ | .6798 | .000 |

Bonferroni P value to correct for multiple comparisons; *. The mean difference is significant at the 0.05 level.

#### *ANOVA - Age*

|  | Sum of Squares | df | Mean Square | F | Sig. |
| --- | --- | --- | --- | --- | --- |
| Between Groups | 228386.027 | 3 | 76128.676 | 163.723 | .000 |
| Within Groups | 11412106.204 | 24543 | 464.984 |  |  |
| Total | 11640492.231 | 24546 |  |  |  |

**Post Hoc Comparisons -** **Age**

| (I) Age | (J) Age | Mean Difference (I-J) | Std. Error | Sig. |
| --- | --- | --- | --- | --- |
| More than 60 years | 0 to 20 years | -2.4450^*^ | .3995 | .000 |
|  | 20 to 40 years | -7.7012^*^ | .3911 | .000 |
|  | 40 to 60 years | -6.3884^*^ | .3981 | .000 |

Bonferroni P value to correct for multiple comparisons; *. The mean difference is significant at the 0.05 level.

#### *ANOVA - BMI*

|  | Sum of Squares | df | Mean Square | F | Sig. |
| --- | --- | --- | --- | --- | --- |
| Between Groups | 26518.677 | 3 | 8839.559 | 18.680 | .000 |
| Within Groups | 11613973.554 | 24543 | 473.209 |  |  |
| Total | 11640492.231 | 24546 |  |  |  |

**Post Hoc Comparisons -** **BMI**

| (I) BMI | (J) BMI | Mean Difference (I-J) | Std. Error | Sig. |
| --- | --- | --- | --- | --- |
| Obesity | Underweight | 2.9100^*^ | .9393 | .012 |
|  | Normal weight | 2.3110^*^ | .3399 | .000 |
|  | Preobesity | 1.7621^*^ | .3387 | .000 |

Bonferroni P value to correct for multiple comparisons; *. The mean difference is significant at the 0.05 level.

#### *ANOVA - Smoking status*

|  | Sum of Squares | df | Mean Square | F | Sig. |
| --- | --- | --- | --- | --- | --- |
| Between Groups | 14946.416 | 3 | 4982.139 | 10.518 | .000 |
| Within Groups | 11625545.816 | 24543 | 473.681 |  |  |
| Total | 11640492.231 | 24546 |  |  |  |

**Post Hoc Comparisons - Smoking status**

| (I) Smoking_status | (J) Smoking_status | Mean Difference (I-J) | Std. Error | Sig. |
| --- | --- | --- | --- | --- |
| Yes | never | 2.1688^*^ | .4343 | .000 |
|  | Quit | 1.3668^*^ | .4926 | .033 |
|  | Vape | .5717 | .6090 | 1.000 |

Bonferroni P value to correct for multiple comparisons; *. The mean difference is significant at the 0.05 level.

#### *ANOVA - Alcohol consumption status*

|  | Sum of Squares | df | Mean Square | F | Sig. |
| --- | --- | --- | --- | --- | --- |
| Between Groups | 45557.171 | 2 | 22778.585 | 48.217 | .000 |
| Within Groups | 11594935.060 | 24544 | 472.414 |  |  |
| Total | 11640492.231 | 24546 |  |  |  |

**Post Hoc Comparisons - Alcohol consumption status**

| (I) Alcohol_consumption | (J) Alcohol_consumption | Mean Difference (I-J) | Std. Error | Sig. |
| --- | --- | --- | --- | --- |
| Have in last 14 days | Never | 3.3148^*^ | .3377 | .000 |
|  | None in last 14 days | .9982^*^ | .3458 | .012 |

Bonferroni P value to correct for multiple comparisons; *. The mean difference is significant at the 0.05 level.

#### *ANOVA - Nonprescription/recreational drugs use status*

|  | Sum of Squares | df | Mean Square | F | Sig. |
| --- | --- | --- | --- | --- | --- |
| Between Groups | 49527.212 | 2 | 24763.606 | 52.524 | .000 |
| Within Groups | 10925864.659 | 23174 | 471.471 |  |  |
| Total | 10975391.871 | 23176 |  |  |  |

**Post Hoc Comparisons - Nonprescription/recreational drugs use status**

| (I) Nonprescription_use | (J) Nonprescription_use | Mean Difference (I-J) | Std. Error | Sig. |
| --- | --- | --- | --- | --- |
| Have in last 28 days | Never | 3.4671^*^ | .3656 | .000 |
|  | None in last 28 days | 1.1801^*^ | .4071 | .011 |

Bonferroni P value to correct for multiple comparisons; *. The mean difference is significant at the 0.05 level.

#### *Independent Samples Test——Underlying medical conditions*

|  | | N | Mean | Std. Deviation | Std. Error Mean |
| --- | --- | --- | --- | --- | --- |
| Opinion_infection | None | 14983 | 31.991 | 21.6003 | .1765 |
|  | Have | 9564 | 35.098 | 21.9177 | .2241 |

|  | | F | Sig. | t | df | Sig.  (2-tailed) | Mean Difference |
| --- | --- | --- | --- | --- | --- | --- | --- |
|  |  |  |  |  |  |  |  |
| Opinion_infection | Equal variances assumed | .830 | .362 | -10.930 | 24545 | .000 | -3.1077 |
|  | Equal variances not assumed |  |  | -10.895 | 20152.155 | .000 | -3.1077 |

#### *Independent Samples Test——Number of close contacts*

|  | | N | Mean | Std. Deviation | Std. Error Mean |
| --- | --- | --- | --- | --- | --- |
| Opinion_infection | Less than 10 people | 19772 | 31.147 | 20.8444 | .1482 |
|  | More than 10 people | 4775 | 41.708 | 23.4322 | .3391 |

|  | | F | Sig. | t | df | Sig.  (2-tailed) | Mean Difference |
| --- | --- | --- | --- | --- | --- | --- | --- |
|  |  |  |  |  |  |  |  |
| Opinion_infection | Equal variances assumed | 120.654 | .000 | -30.645 | 24545 | .000 | -10.5608 |
|  | Equal variances not assumed |  |  | -28.536 | 6713.835 | .000 | -10.5608 |

#### *Independent Samples Test——Number of cohabitants*

|  | | N | Mean | Std. Deviation | Std. Error Mean |
| --- | --- | --- | --- | --- | --- |
| Opinion_infection | Less than 5 people | 22741 | 33.182 | 21.7468 | .1442 |
|  | More than 5 people | 1806 | 33.450 | 22.1561 | .5214 |

|  | | F | Sig. | t | df | Sig.  (2-tailed) | Mean Difference |
| --- | --- | --- | --- | --- | --- | --- | --- |
|  |  |  |  |  |  |  |  |
| Opinion_infection | Equal variances assumed | 1.053 | .305 | -.503 | 24545 | .615 | -.2679 |
|  | Equal variances not assumed |  |  | -.495 | 2090.794 | .620 | -.2679 |

#### *ANOVA - Text_working*

|  | Sum of Squares | df | Mean Square | F | Sig. |
| --- | --- | --- | --- | --- | --- |
| Between Groups | 493682.500 | 4 | 123420.625 | 271.736 | .000 |
| Within Groups | 11146809.731 | 24542 | 454.193 |  |  |
| Total | 11640492.231 | 24546 |  |  |  |

**Post Hoc Comparisons - Text_working**

| (I) Text_working | (J) Text_working | Mean Difference (I-J) | Std. Error | Sig. |
| --- | --- | --- | --- | --- |
| Travel non critical | Home | 2.3708^*^ | .7420 | .014 |
|  | Never | 5.8538^*^ | .5707 | .000 |
|  | Stopped | 2.3226^*^ | .5276 | .000 |
|  | Travel critical | -6.8870^*^ | .5609 | .000 |

Bonferroni P value to correct for multiple comparisons; *. The mean difference is significant at the 0.05 level.

#### *ANOVA - Take steps to reduce my risk*

|  | Sum of Squares | df | Mean Square | F | Sig. |
| --- | --- | --- | --- | --- | --- |
| Between Groups | 5174.602 | 2 | 2587.301 | 5.458 | .004 |
| Within Groups | 11635317.629 | 24544 | 474.060 |  |  |
| Total | 11640492.231 | 24546 |  |  |  |

**Post Hoc Comparisons - Take steps to reduce my risk**

| (I) steps_reduce_my_risk | (J) steps_reduce_my_risk | Mean Difference (I-J) | Std. Error | Sig. |
| --- | --- | --- | --- | --- |
| Agree | Disagree | 1.9377 | 1.1030 | .237 |
|  | Neutral | 1.2013^*^ | .4170 | .012 |

Bonferroni P value to correct for multiple comparisons; *. The mean difference is significant at the 0.05 level.

#### *ANOVA - Cohabitants take steps to reduce risk*

|  | Sum of Squares | df | Mean Square | F | Sig. |
| --- | --- | --- | --- | --- | --- |
| Between Groups | 22403.792 | 2 | 11201.896 | 23.665 | .000 |
| Within Groups | 11618088.440 | 24544 | 473.358 |  |  |
| Total | 11640492.231 | 24546 |  |  |  |

**Post Hoc Comparisons - Cohabitants take steps to reduce risk**

| (I) Cohabitants_steps_reduce_risk | (J) Cohabitants_steps_reduce_risk | Mean Difference (I-J) | Std. Error | Sig. |
| --- | --- | --- | --- | --- |
|  |  |  |  |  |
| Agree | Disagree | -5.2134^*^ | .8153 | .000 |
|  | Neutral | -1.0752^*^ | .3473 | .006 |

Bonferroni P value to correct for multiple comparisons; *. The mean difference is significant at the 0.05 level.

#### *ANOVA - Wear a mask*

|  | Sum of Squares | df | Mean Square | F | Sig. |
| --- | --- | --- | --- | --- | --- |
| Between Groups | 46366.317 | 2 | 23183.158 | 49.077 | .000 |
| Within Groups | 11594125.914 | 24544 | 472.381 |  |  |
| Total | 11640492.231 | 24546 |  |  |  |

**Post Hoc Comparisons - Wear a mask**

| (I) Wear_a_mask | (J) Wear_a_mask | Mean Difference (I-J) | Std. Error | Sig. |
| --- | --- | --- | --- | --- |
| Usually | Rarely | 2.2453^*^ | .3454 | .000 |
|  | Sometimes | -.9662 | .4249 | .069 |

Bonferroni P value to correct for multiple comparisons; *. The mean difference is significant at the 0.05 level.

### Environmental factors regarding COVID-19 stratified by demographic variables

| **Demographics** | **Environmental factors, n (%) or mean (standard deviation)** | | | | | | | | |
| --- | --- | --- | --- | --- | --- | --- | --- | --- | --- |
|  | **Number of close contacts** | | **Number of cohabitants** | | **Text_working** | | | | |
|  | ≤10 people | >10 people | ≤5 people | > 5 people | Home | Never | Stopped | Travel critical | Travel non critical |
| Opinion_infection (mean±SD) | 31.15± 20.84 | 41.71± 23.43*** | 33.18± 21.75 | 33.45± 22.16 | 31.55± 21.36 | 28.07± 20.82 | 31.60± 20.49 | 40.81± 23.05 | 33.92± 21.56*** |
| **Gender** |  |  |  |  |  |  |  |  |  |
| Female | 10042 (80.6) | 2423 (19.4) | 11522 (92.4) | 943  (7.6) | 729  (5.8) | 2881  (23.1) | 5356  (43.0) | 2710 (21.7) | 789  (6.3) |
| Male | 87056 (80.5) | 24669 (19.5) | 11219 (92.9) | 863  (7.1) | 710  (5.9) | 2122  (17.6) | 5113  (42.6) | 2993  (24.8) | 1144  (9.5) *** |
| **Place of current residence** | | |  |  |  |  |  |  |  |
| 0 to1000 cases | 511  (84.3) | 95  (15.7) | 559  (92.2) | 47  (7.8) | 27 (4.5) | 140 (23.1) | 264 (43.6) | 142 (23.4) | 33  (5.4) |
| 1001 to 5000 cases | 4162  (78.1) | 1164  (21.9) | 4923 (92.4) | 403  (7.6) | 315 (5.9) | 1057 (19.8) | 2099 (39.4) | 1359 (25.5) | 496  (9.3) |
| 5001 to 10000 cases | 2842  (78.1) | 796  (21.9) | 3373 (92.7) | 265  (7.3) | 227 (6.2) | 763 (21.0) | 1442 (39.6) | 899 (24.7) | 307  (8.4) |
| 10001 to 20000 cases | 5592  (80.6) | 1345  (19.4) | 6410 (92.4) | 527  (7.6) | 429 (6.2) | 1384 (20.0) | 3009 (43.4) | 1578 (22.7) | 537  (7.7) |
| 20001 to 40000 cases | 5630  (82.4) | 1204  (17.6) | 6349 (92.9) | 485  (7.1) | 367 (5.4) | 1429 (20.9) | 3072 (45.0) | 1460 (21.4) | 506  (7.4) |
| 40001 or more cases | 1035  (85.8) | 171  (14.2)*** | 1127 (93.4) | 79  (6.6) | 74 (6.1) | 230 (19.1) | 583 (48.3) | 265 (22.0) | 54  (4.5)*** |
| **Age** |  |  |  |  |  |  |  |  |  |
| 0 to 20 years | 4718 (77.3) | 1382 (22.7) | 5244 (86.0) | 856  (14.0) | 273 (4.5) | 827 (13.6) | 3409  (55.9) | 1058 (17.3) | 533  (8.7) |
| 20 to 40 years | 4949 (74.1) | 1728 (25.9) | 6217 (93.1) | 460  (6.9) | 370 (5.5) | 659 (9.9) | 2823 (42.3) | 2199 (32.9) | 626  (9.4) |
| 40 to 60 years | 4989 (80.6) | 1202 (19.4) | 5807 (93.8) | 384  (6.2) | 486 (7.9) | 786 (12.7) | 2641 (42.7) | 1755 (28.3) | 523  (8.4) |
| More than 60 years | 5116 (91.7) | 463 (8.3)*** | 5473 (98.1) | 106 (1.9)*** | 310 (5.6) | 2731 (49.0) | 1596  (28.6) | 691 (12.4) | 251 (4.5)*** |
| **BMI** |  |  |  |  |  |  |  |  |  |
| Underweight | 467 (82.2) | 101 (17.8) | 510  (89.8) | 58  (10.2) | 28 (4.9) | 81 (14.3) | 314 (55.3) | 90 (15.8) | 55  (9.7) |
| Normal weight | 5840 (81.8) | 1296 (18.2) | 6542 (91.7) | 594  (8.3) | 411 (5.8) | 1354 (19.0) | 3443 (48.2) | 1436 (20.1) | 492  (6.9) |
| Preobesity | 5868 (81.2) | 1358 (18.8) | 6760 (93.6) | 466  (6.4) | 421 (5.8) | 1503 (20.8) | 3008 (41.6) | 1715 (23.7) | 579  (8.0) |
| Obesity | 7597 (79.0) | 2020 (21.0)*** | 8929 (92.8) | 688 (7.2)*** | 579 (6.0) | 2065 (21.5) | 3704 (38.5) | 2462 (25.6) | 807 (8.4)*** |
| **Smoking status** |  |  |  |  |  |  |  |  |  |
| Never | 11425  (82.1) | 2494  (17.9) | 12882  (92.5) | 1037  (7.5) | 858 (6.2) | 2681 (19.3) | 6398 (46.0) | 2996 (21.5) | 986  (7.1) |
| Quit | 4483  (83.4) | 891  (16.6) | 5092  (94.8) | 282  (5.2) | 327  (6.1) | 1428 (26.6) | 2040 (38.0) | 1226 (22.8) | 353  (6.6) |
| Vape | 1617  (73.9) | 572  (26.1) | 1964  (89.7) | 225  (10.3) | 104 (4.8) | 296 (13.5) | 989 (45.2) | 558 (25.5) | 242  (11.1) |
| Yes | 2247  (73.3) | 818  (26.7)*** | 2803  (91.5) | 262  (8.5) *** | 150  (4.9) | 598 (19.5) | 1042  (34.0) | 923 (30.1) | 352 (11.5)*** |
| **Alcohol consumption status** | | |  |  |  |  |  |  |  |
| Never | 5168  (83.8) | 997  (16.2) | 5559 (90.2) | 606  (9.8) | 347 (5.6) | 1573 (25.5) | 2717 (44.1) | 1076 (17.5) | 452  (7.3) |
| None in last 14 days | 4527  (78.7) | 1223  (21.3) | 5274 (91.7) | 476  (8.3) | 316  (5.5) | 1220 (21.2) | 2297 (39.9) | 1467 (25.5) | 450  (7.8) |
| Some in last 14 days | 10077  (79.8) | 2555  (20.2)*** | 11908 (94.3) | 724 (5.7)*** | 776 (6.1) | 2210 (17.5) | 5455 (43.2) | 3160 (25.0) | 1031 (8.2)*** |
| **Nonprescription/recreational drugs use status** | | | | | | | | | |
| Never | 9452 (81.3) | 2169 (18.7) | 1072 (92.3) | 896  (7.7) | 676  (5.8) | 2510  (21.6) | 4889  (42.1) | 2719  (23.4) | 827  (7.1) |
| None in last 28 days | 5210  (80.3) | 1281  (19.7) | 6070 (93.5) | 421  (6.5) | 405 (6.2) | 1223 (18.8) | 2782 (42.9) | 1561 (24.0) | 520  (8.0) |
| Some in last 28 days | 3966 (78.3) | 1099 (21.7)*** | 4647 (91.7) | 418  (8.3)** | 285 (5.6) | 875 (17.3) | 2285 (45.1) | 1121 (22.1) | 499 (9.9)*** |
| **Underlying medical conditions** | | |  |  |  |  |  |  |  |
| None | 11843 (79.0) | 3140 (21.0) | 13812 (92.2) | 1171  (7.8) | 890  (5.9) | 2422  (16.2) | 6626  (44.2) | 3743  (25.0) | 1302  (8.7) |
| Have | 7929  (82.9) | 1635  (17.1)*** | 8919 (93.4) | 635  (6.6) ** | 549  (5.7) | 2581  (27.0) | 3843  (40.2) | 1960  (20.5) | 631  (6.6) *** |

Note: ***P<0.001; **P<0.01; *P<0.05

### Follow-up analysis for Table 4 in the main document

Table 4 is the results of binary logistic regression analysis of factors significantly associated with environmental factors

The environmental factors include close contact with more than 10 people, more than 5 people living together and stop going to work/school

#### *The results of binary logistic regression analysis with more than 10 people in close contact*

**Model Summary**

| Step | -2 Log likelihood | Cox & Snell R Square | Nagelkerke R Square |
| --- | --- | --- | --- |
| 1 | 22388.439^a^ | .071 | .113 |

a. Estimation terminated at iteration number 5 because parameter estimates changed by less than .001.

**Hosmer and Lemeshow Test**

| Step | Chi-square | df | Sig. |
| --- | --- | --- | --- |
| 1 | 13.553 | 8 | .094 |

**Classification Table^a^**

| Observed | | | Predicted | | |
| --- | --- | --- | --- | --- | --- |
|  |  |  | contact_more_than_10 | | Percentage Correct |
|  |  |  | 0 | 1 |  |
| Step 1 | contact_more_than_10 | 0 | 19647 | 125 | 99.4 |
|  |  | 1 | 4627 | 148 | 3.1 |
|  | Overall Percentage | |  |  | 80.6 |

a. The cut value is .500

**Variables in the Equation**

|  | | B | S.E. | Sig. | Exp(B) | 95% C.I.for EXP(B) | |
| --- | --- | --- | --- | --- | --- | --- | --- |
|  |  |  |  |  |  | Lower | Upper |
| Step 1^a^ | Opinion_infection | .021 | .001 | .000 | 1.021 | 1.020 | 1.023 |
|  | female | -.043 | .034 | .208 | .958 | .897 | 1.024 |
|  | 0 to1000 cases | .224 | .144 | .118 | 1.251 | .944 | 1.658 |
|  | 1001 to 5000 cases | .598 | .092 | .000 | 1.819 | 1.519 | 2.178 |
|  | 5001 to 10000 cases | .638 | .095 | .000 | 1.892 | 1.571 | 2.279 |
|  | 10001 to 20000 cases | .459 | .091 | .000 | 1.582 | 1.324 | 1.891 |
|  | 20001 to 40000 cases | .369 | .091 | .000 | 1.446 | 1.209 | 1.729 |
|  | 0 to 20 years | 1.174 | .063 | .000 | 3.235 | 2.861 | 3.657 |
|  | 20 to 40 years | 1.135 | .059 | .000 | 3.110 | 2.771 | 3.491 |
|  | 40 to 60 years | .787 | .060 | .000 | 2.196 | 1.953 | 2.470 |
|  | underweight | -.449 | .118 | .000 | .638 | .507 | .804 |
|  | normal_weight | -.248 | .043 | .000 | .780 | .718 | .848 |
|  | preobesity | -.098 | .041 | .018 | .907 | .836 | .983 |
|  | never_smoking | -.509 | .051 | .000 | .601 | .544 | .665 |
|  | quit | -.452 | .058 | .000 | .637 | .568 | .713 |
|  | vape | -.178 | .067 | .008 | .837 | .733 | .955 |
|  | never_drinking | -.254 | .046 | .000 | .776 | .709 | .849 |
|  | none_14days | .067 | .041 | .108 | 1.069 | .986 | 1.159 |
|  | never_drugs | .196 | .045 | .000 | 1.216 | 1.113 | 1.328 |
|  | none_28days | .054 | .047 | .244 | 1.056 | .963 | 1.157 |
|  | have_diseases | -.170 | .037 | .000 | .843 | .785 | .906 |
|  | Constant | -2.989 | .118 | .000 | .050 |  |  |

#### *The results of binary logistic regression analysis with more than 5 people living together*

**Model Summary**

| Step | -2 Log likelihood | Cox & Snell R Square | Nagelkerke R Square |
| --- | --- | --- | --- |
| 1 | 12162.388^a^ | .030 | .073 |

a. Estimation terminated at iteration number 7 because parameter estimates changed by less than .001.

**Hosmer and Lemeshow Test**

| Step | Chi-square | df | Sig. |
| --- | --- | --- | --- |
| 1 | 5.094 | 8 | .748 |

**Classification Table^a^**

| Observed | | | Predicted | | |
| --- | --- | --- | --- | --- | --- |
|  |  |  | cohabitant_more_than_5 | | Percentage Correct |
|  |  |  | 0 | 1 |  |
| Step 1 | cohabitant_more_than_5 | 0 | 22741 | 0 | 100.0 |
|  |  | 1 | 1806 | 0 | .0 |
|  | Overall Percentage | |  |  | 92.6 |

a. The cut value is .500

**Variables in the Equation**

|  | | B | S.E. | Sig. | Exp(B) | 95% C.I.for EXP(B) | |
| --- | --- | --- | --- | --- | --- | --- | --- |
|  |  |  |  |  |  | Lower | Upper |
| Step 1^a^ | Opinion_infection | .000 | .001 | .839 | 1.000 | .998 | 1.003 |
|  | female | .096 | .050 | .054 | 1.101 | .998 | 1.215 |
|  | 0 to1000 cases | .121 | .195 | .534 | 1.129 | .771 | 1.653 |
|  | 1001 to 5000 cases | .079 | .129 | .540 | 1.082 | .840 | 1.394 |
|  | 5001 to 10000 cases | .082 | .135 | .540 | 1.086 | .834 | 1.414 |
|  | 10001 to 20000 cases | .114 | .127 | .366 | 1.121 | .875 | 1.437 |
|  | 20001 to 40000 cases | .066 | .127 | .607 | 1.068 | .832 | 1.371 |
|  | 0 to 20 years | 2.101 | .110 | .000 | 8.175 | 6.592 | 10.140 |
|  | 20 to 40 years | 1.384 | .112 | .000 | 3.991 | 3.203 | 4.972 |
|  | 40 to 60 years | 1.237 | .113 | .000 | 3.446 | 2.763 | 4.297 |
|  | underweight | -.199 | .148 | .181 | .820 | .613 | 1.097 |
|  | normal_weight | -.062 | .062 | .317 | .940 | .833 | 1.061 |
|  | preobesity | -.054 | .064 | .398 | .948 | .836 | 1.074 |
|  | never_smoking | -.319 | .078 | .000 | .727 | .623 | .847 |
|  | quit | -.297 | .091 | .001 | .743 | .621 | .889 |
|  | vape | -.193 | .099 | .051 | .824 | .679 | 1.001 |
|  | never_drinking | .322 | .065 | .000 | 1.379 | 1.214 | 1.567 |
|  | none_14days | .265 | .063 | .000 | 1.303 | 1.151 | 1.475 |
|  | never_drugs | .119 | .067 | .076 | 1.127 | .987 | 1.286 |
|  | none_28days | .008 | .072 | .914 | 1.008 | .876 | 1.160 |
|  | have_diseases | .050 | .054 | .356 | 1.051 | .946 | 1.168 |
|  | Constant | -4.027 | .182 | .000 | .018 |  |  |

#### *The results of binary logistic regression analysis of stopping work/school*

**Model Summary**

| Step | -2 Log likelihood | Cox & Snell R Square | Nagelkerke R Square |
| --- | --- | --- | --- |
| 1 | 32068.846^a^ | .057 | .076 |

a. Estimation terminated at iteration number 4 because parameter estimates changed by less than .001.

**Hosmer and Lemeshow Test**

| Step | Chi-square | df | Sig. |
| --- | --- | --- | --- |
| 1 | 46.477 | 8 | .000 |

**Classification Table^a^**

| Observed | | | Predicted | | |
| --- | --- | --- | --- | --- | --- |
|  |  |  | Stopped | | Percentage Correct |
|  |  |  | 0 | 1 |  |
| Step 1 | Stopped | 0 | 11144 | 2934 | 79.2 |
|  |  | 1 | 6451 | 4018 | 38.4 |
|  | Overall Percentage | |  |  | 61.8 |

a. The cut value is .500

**Variables in the Equation**

|  | | B | S.E. | Sig. | Exp(B) | 95% C.I.for EXP(B) | |
| --- | --- | --- | --- | --- | --- | --- | --- |
|  |  |  |  |  |  | Lower | Upper |
| Step 1^a^ | Opinion_infection | -.007 | .001 | .000 | .993 | .992 | .994 |
|  | female | .100 | .027 | .000 | 1.105 | 1.049 | 1.165 |
|  | 0 to1000 cases | -.220 | .103 | .033 | .803 | .656 | .983 |
|  | 1001 to 5000 cases | -.415 | .066 | .000 | .660 | .580 | .751 |
|  | 5001 to 10000 cases | -.390 | .069 | .000 | .677 | .592 | .775 |
|  | 10001 to 20000 cases | -.253 | .064 | .000 | .776 | .684 | .881 |
|  | 20001 to 40000 cases | -.179 | .064 | .006 | .836 | .737 | .949 |
|  | 0 to 20 years | 1.221 | .044 | .000 | 3.389 | 3.110 | 3.693 |
|  | 20 to 40 years | .697 | .041 | .000 | 2.009 | 1.853 | 2.177 |
|  | 40 to 60 years | .717 | .041 | .000 | 2.049 | 1.892 | 2.219 |
|  | underweight | .393 | .091 | .000 | 1.481 | 1.240 | 1.770 |
|  | normal_weight | .272 | .034 | .000 | 1.313 | 1.230 | 1.403 |
|  | preobesity | .142 | .033 | .000 | 1.152 | 1.080 | 1.229 |
|  | never_smoking | .538 | .045 | .000 | 1.712 | 1.569 | 1.868 |
|  | quit | .354 | .049 | .000 | 1.424 | 1.293 | 1.569 |
|  | vape | .162 | .060 | .007 | 1.176 | 1.046 | 1.323 |
|  | never_drinking | -.167 | .035 | .000 | .846 | .789 | .907 |
|  | none_14days | -.209 | .034 | .000 | .811 | .759 | .867 |
|  | never_drugs | -.123 | .035 | .001 | .884 | .825 | .948 |
|  | none_28days | .022 | .037 | .558 | 1.022 | .950 | 1.100 |
|  | have_diseases | .087 | .029 | .003 | 1.091 | 1.031 | 1.154 |
|  | Constant | -.941 | .086 | .000 | .390 |  |  |

### Behavioral factors regarding COVID-19 stratified by demographic variables

| **Demographics** | **Behaviors, n (%) or mean (standard deviation)** | | | | | | | | |
| --- | --- | --- | --- | --- | --- | --- | --- | --- | --- |
|  | **Participants taking steps to reduce their risk** | | | **Participants’ cohabitants taking steps to reduce their** | | | **Wearing a mask** | | |
|  | Disagree | Neutral | Agree | Disagree | Neutral | Agree | Rarely | Some-times | Usually |
| Opinion_infection (mean±SD) | 31.45 (25.89) | 32.19  (22.17) | 33.39 (21.63)** | 38.04 (24.01) | 33.90 (21.75) | 32.83 (21.67)*** | 32.05 (21.66) | 35.26 (21.42) | 34.29 (22.21)*** |
| **Gender** |  |  |  |  |  |  |  |  |  |
| Female | 150  (1.2) | 1432  (11.5) | 10883  (87.3) | 422  (3.4) | 2644  (21.2) | 9399  (75.4) | 6663 (53.5) | 2683 (21.5) | 3119 (25.0) |
| Male | 247  (2.0) | 1701  (14.1) | 10134  (83.9)*** | 318  (2.6) | 2312 (19.1) | 9452 (78.2)*** | 7390 (61.2) | 2298 (19.0) | 2394 (19.8)*** |
| **Place of current residence** | | | | | | | | | |
| 0 to 1000 cases | 13  (2.1) | 82  (13.5) | 511  (84.3) | 23  (3.8) | 112  (18.5) | 471  (77.7) | 345 (56.9) | 122  (20.1) | 139  (22.9) |
| 1001 to 5000 cases | 94  (1.8) | 709  (13.3) | 4523  (84.9) | 172  (3.2) | 1131  (21.2) | 4023  (75.5) | 3332 (62.6) | 1036 (19.5) | 958  (18.0) |
| 5001 to 10000 cases | 63  (1.7) | 446  (12.3) | 3129  (86.0) | 95  (2.6) | 711  (19.5) | 2832  (77.8) | 2185  (60.1) | 750 (20.6) | 703  (19.3) |
| 10001to 20000 cases | 105  (1.5) | 902  (13.0) | 5930  (85.5) | 221  (3.2) | 1384  (20.0) | 5332  (76.9) | 4036 (58.2) | 1401 (20.2) | 1500 (21.6) |
| 20001 to 40000 cases | 107  (1.6) | 832  (12.2) | 5895  (86.3) | 191  (2.8) | 1372  (20.1) | 5271  (77.1) | 3596 (52.6) | 1407 (20.6) | 1831 (26.8) |
| 40001 or more cases | 15  (1.2) | 162  (13.4) | 1029  (85.3) | 38  (3.2) | 246  (20.4) | 922  (76.5) | 559 (46.4) | 265 (22.0) | 382 (31.7)*** |
| **Age** |  |  |  |  |  |  |  |  |  |
| 0 to 20 years | 225  (3.7) | 1305 (21.4) | 4570 (74.9) | 363  (6.0) | 1379 (22.6) | 4358 (71.4) | 4290 (70.3) | 944 (15.5) | 866  (14.2) |
| 20 to 40 years | 90  (1.3) | 831  (12.4) | 5756  (86.2) | 201  (3.0) | 1449 (21.7) | 5027 (75.3) | 4175 (62.5) | 1329 (19.9) | 1173 (17.6) |
| 40 to 60 years | 39  (0.6) | 543  (8.8) | 5609 (90.6) | 85  (1.4) | 1128 (18.2) | 4978 (80.4) *** | 3284 (53.0) | 1379 (22.3) | 1528 (24.7) *** |
| More than 60 years | 43  (0.8) | 454  (8.1) | 5082 (91.1)*** | 91  (1.6) | 1000 (17.9) | 4488 (80.4)*** | 2304 (41.3) | 1329 (23.8) | 1946 (34.9)*** |
| **BMI** |  |  |  |  |  |  |  |  |  |
| Underweight | 21  (3.7) | 114  (20.1) | 433  (76.2) | 34  (6.0) | 114 (20.1) | 420  (73.9) | 378 (66.5) | 86 (15.1) | 104  (18.3) |
| Normal weight | 153  (2.1) | 975  (13.7) | 6008  (84.2) | 275  (3.9) | 1409 (19.7) | 5452 (76.4) | 4172 (58.5) | 1376 (19.3) | 1588 (22.3) |
| Preobesity | 81  (1.1) | 858  (11.9) | 6287  (87.0) | 159  (2.2) | 1403 (19.4) | 5664 (78.4) | 4036 (55.9) | 1528 (21.1) | 1662 (23.0) |
| Obesity | 142  (1.5) | 1186  (12.3) | 8289  (86.2)*** | 272  (2.8) | 2030 (21.1) | 7315 (76.1)*** | 5467 (56.8) | 1991 (20.7) | 2159 (22.4)*** |
| **Smoking status** |  |  |  |  |  |  |  |  |  |
| Never | 170  (1.2) | 1619  (11.6) | 12130  (87.1) | 378  (2.7) | 2640 (19.0) | 10901 (78.3) | 8052 (57.8) | 2829 (20.3) | 3038 (21.8) |
| Quit | 65  (1.2) | 573  (10.7) | 4736  (88.1) | 137  (2.5) | 1054 (19.6) | 4183 (77.8) | 2787 (51.9) | 1117 (20.8) | 1470 (27.4) |
| Vape | 80  (3.7) | 443  (20.2) | 1666  (76.1) | 92  (4.2) | 507  (23.2) | 1590  (72.6) | 1413 (64.6) | 409 (18.7) | 367  (16.8) |
| Yes | 82  (2.7) | 498  (16.2) | 2485  (81.1)*** | 133  (4.3) | 755  (24.6) | 2177  (71.0) *** | 1801 (58.8) | 626 (20.4) | 638 (20.8)*** |
| **Alcohol consumption status** | | | | | | | | | |
| Never | 97  (1.6) | 873  (14.2) | 5195  (84.3) | 218  (3.5) | 1227  (19.9) | 4720  (76.6) | 3539  (57.4) | 1123  (18.2) | 1503  (24.4) |
| None in last 14 days | 94  (1.6) | 767  (13.3) | 4889  (85.0) | 201  (3.5) | 1203  (20.9) | 4346  (75.6) | 3335  (58.0) | 1124  (19.5) | 1291  (22.5) |
| Some in last 14 days | 206  (1.6) | 1493  (11.8) | 10933  (86.6)*** | 321  (2.5) | 2526  (20.0) | 9785  (77.5)*** | 7179 (56.8) | 2734 (21.6) | 2719 (21.5)*** |
| **Nonprescription/recreational drugs use status** | | | | | | | | | |
| Never | 144  (1.2) | 1395  (12.0) | 10082  (86.8) | 287  (2.5) | 2230  (19.2) | 9104  (78.3) | 6715  (57.8) | 2305  (19.8) | 2601  (22.4) |
| None in last 28 days | 93  (1.4) | 754  (11.6) | 5644  (87.0) | 183  (2.8) | 1295 (20.0) | 5013 (77.2) | 3734 (57.5) | 1321 (20.4) | 1436 (22.1) |
| Some in last 28 days | 145  (2.9) | 774  (15.3) | 4146  (81.9)*** | 237  (4.7) | 1142  (22.5) | 3686  (72.8)*** | 2927  (57.8) | 1054  (20.8) | 1084  (21.4) |
| **Underlying medical conditions** | | | | | | | | | |
| None | 274  (1.8) | 2016  (13.5) | 12693  (84.7) | 433  (2.4) | 3004 (20.0) | 11546 (77.1) | 9118  (60.9) | 2938  (19.6) | 2927  (19.5) |
| Have | 123  (1.3) | 1117  (11.7) | 8324  (87.0)*** | 307  (3.2) | 1952  (20.4) | 7305  (76.4)*** | 4935 (51.6) | 2043 (21.4) | 2586 (27.0)*** |

Note: ***P<0.001; **P<0.01; *P<0.05

### Follow-up analysis for Table 5 in the main document

Table 5 is the results of binary logistic regression analysis of significant behavioral factors

The behavioral factors include disagreeing with themselves or their cohabitants to take steps to reduce the risk of COVID-19, and rarely wearing masks.

#### *Disagree with taking steps to reduce the risk of getting COVID-19*

**Model Summary**

| Step | -2 Log likelihood | Cox & Snell R Square | Nagelkerke R Square |
| --- | --- | --- | --- |
| 1 | 3754.739^a^ | .012 | .082 |

a. Estimation terminated at iteration number 8 because parameter estimates changed by less than .001.

**Hosmer and Lemeshow Test**

| Step | Chi-square | df | Sig. |
| --- | --- | --- | --- |
| 1 | 15.459 | 8 | .051 |

**Classification Table^a^**

| Observed | | | Predicted | | |
| --- | --- | --- | --- | --- | --- |
|  |  |  | no_steps_reduce_my_risk | | Percentage Correct |
|  |  |  | .00 | 1.00 |  |
| Step 1 | no_steps_reduce_my_risk | .00 | 24150 | 0 | 100.0 |
|  |  | 1.00 | 397 | 0 | .0 |
|  | Overall Percentage | |  |  | 98.4 |

a. The cut value is .500

**Variables in the Equation**

|  | | B | S.E. | Sig. | Exp(B) | 95% C.I.for EXP(B) | |
| --- | --- | --- | --- | --- | --- | --- | --- |
|  |  |  |  |  |  | Lower | Upper |
| Step 1^a^ | Opinion_infection | -.003 | .002 | .225 | .997 | .992 | 1.002 |
|  | female | -.508 | .106 | .000 | .602 | .489 | .741 |
|  | 0 to1000 cases | .564 | .386 | .144 | 1.758 | .825 | 3.746 |
|  | 1001 to 5000 cases | .275 | .282 | .330 | 1.316 | .757 | 2.288 |
|  | 5001 to 10000 cases | .289 | .292 | .321 | 1.335 | .754 | 2.365 |
|  | 10001 to 20000 cases | .140 | .280 | .618 | 1.150 | .664 | 1.989 |
|  | 20001 to 40000 cases | .175 | .280 | .530 | 1.192 | .689 | 2.061 |
|  | 0 to 20 years | 1.482 | .183 | .000 | 4.402 | 3.078 | 6.295 |
|  | 20 to 40 years | .390 | .194 | .044 | 1.477 | 1.010 | 2.159 |
|  | 40 to 60 years | -.279 | .225 | .215 | .757 | .487 | 1.176 |
|  | underweight | .066 | .247 | .789 | 1.068 | .659 | 1.732 |
|  | normal_weight | -.027 | .123 | .829 | .974 | .765 | 1.240 |
|  | preobesity | -.376 | .142 | .008 | .686 | .519 | .907 |
|  | never_smoking | -.814 | .150 | .000 | .443 | .330 | .595 |
|  | quit | -.539 | .174 | .002 | .583 | .415 | .820 |
|  | vape | -.275 | .166 | .099 | .760 | .549 | 1.053 |
|  | never_drinking | -.279 | .141 | .048 | .757 | .574 | .998 |
|  | none_14days | -.158 | .129 | .221 | .854 | .662 | 1.100 |
|  | never_drugs | -.233 | .137 | .089 | .792 | .606 | 1.036 |
|  | none_28days | -.211 | .137 | .125 | .810 | .619 | 1.060 |
|  | have_diseases | -.116 | .115 | .311 | .890 | .711 | 1.115 |
|  | Constant | -3.691 | .348 | .000 | .025 |  |  |

#### *Cohabitants disagree with taking steps to reduce the risk of getting COVID-19*

**Model Summary**

| Step | -2 Log likelihood | Cox & Snell R Square | Nagelkerke R Square |
| --- | --- | --- | --- |
| 1 | 6253.812^a^ | .016 | .066 |

a. Estimation terminated at iteration number 7 because parameter estimates changed by less than .001.

**Hosmer and Lemeshow Test**

| Step | Chi-square | df | Sig. |
| --- | --- | --- | --- |
| 1 | 4.016 | 8 | .856 |

**Classification Table^a^**

| Observed | | | Predicted | | |
| --- | --- | --- | --- | --- | --- |
|  |  |  | no_Cohabitants_steps_reduce_risk | | Percentage Correct |
|  |  |  | .00 | 1.00 |  |
| Step 1 | no_Cohabitants_steps_reduce_risk | .00 | 23807 | 0 | 100.0 |
|  |  | 1.00 | 740 | 0 | .0 |
|  | Overall Percentage | |  |  | 97.0 |

a. The cut value is .500

**Variables in the Equation**

|  | | B | S.E. | Sig. | Exp(B) | 95% C.I.for EXP(B) | |
| --- | --- | --- | --- | --- | --- | --- | --- |
|  |  |  |  |  |  | Lower | Upper |
| Step 1^a^ | Opinion_infection | .010 | .002 | .000 | 1.010 | 1.006 | 1.013 |
|  | female | .261 | .077 | .001 | 1.298 | 1.117 | 1.508 |
|  | 0 to1000 cases | .187 | .272 | .492 | 1.205 | .707 | 2.054 |
|  | 1001 to 5000 cases | -.054 | .184 | .769 | .947 | .660 | 1.359 |
|  | 5001 to 10000 cases | -.203 | .197 | .303 | .816 | .555 | 1.201 |
|  | 10001 to 20000 cases | -.011 | .180 | .949 | .989 | .694 | 1.408 |
|  | 20001 to 40000 cases | -.141 | .182 | .438 | .868 | .607 | 1.241 |
|  | 0 to 20 years | 1.336 | .129 | .000 | 3.802 | 2.956 | 4.891 |
|  | 20 to 40 years | .615 | .133 | .000 | 1.850 | 1.425 | 2.402 |
|  | 40 to 60 years | -.179 | .154 | .244 | .836 | .618 | 1.131 |
|  | underweight | .298 | .194 | .124 | 1.348 | .922 | 1.970 |
|  | normal_weight | .139 | .091 | .127 | 1.149 | .961 | 1.374 |
|  | preobesity | -.186 | .103 | .071 | .831 | .679 | 1.016 |
|  | never_smoking | -.464 | .112 | .000 | .629 | .505 | .783 |
|  | quit | -.296 | .128 | .021 | .744 | .579 | .957 |
|  | vape | -.510 | .144 | .000 | .601 | .453 | .796 |
|  | never_drinking | .192 | .100 | .055 | 1.212 | .996 | 1.474 |
|  | none_14days | .182 | .094 | .054 | 1.199 | .997 | 1.443 |
|  | never_drugs | -.364 | .099 | .000 | .695 | .572 | .844 |
|  | none_28days | -.209 | .101 | .039 | .812 | .666 | .990 |
|  | have_diseases | .265 | .080 | .001 | 1.304 | 1.115 | 1.524 |
|  | Constant | -4.144 | .243 | .000 | .016 |  |  |

#### *Rarely wear a mask*

**Model Summary**

| Step | -2 Log likelihood | Cox & Snell R Square | Nagelkerke R Square |
| --- | --- | --- | --- |
| 1 | 31833.475^a^ | .066 | .089 |

a. Estimation terminated at iteration number 4 because parameter estimates changed by less than .001.

**Hosmer and Lemeshow Test**

| Step | Chi-square | df | Sig. |
| --- | --- | --- | --- |
| 1 | 8.871 | 8 | .353 |

**Classification Table^a^**

| Observed | | | Predicted | | |
| --- | --- | --- | --- | --- | --- |
|  |  |  | rarely_wear | | Percentage Correct |
|  |  |  | .00 | 1.00 |  |
| Step 1 | rarely_wear | .00 | 4350 | 6144 | 41.5 |
|  |  | 1.00 | 3077 | 10976 | 78.1 |
|  | Overall Percentage | |  |  | 62.4 |

a. The cut value is .500

**Variables in the Equation**

|  | | B | S.E. | Sig. | Exp(B) | 95% C.I.for EXP(B) | |
| --- | --- | --- | --- | --- | --- | --- | --- |
|  |  |  |  |  |  | Lower | Upper |
| Step 1^a^ | Opinion_infection | -.007 | .001 | .000 | .994 | .992 | .995 |
|  | female | -.289 | .027 | .000 | .749 | .710 | .789 |
|  | 0 to1000 cases | .463 | .104 | .000 | 1.589 | 1.296 | 1.947 |
|  | 1001 to 5000 cases | .678 | .066 | .000 | 1.970 | 1.730 | 2.244 |
|  | 5001 to 10000 cases | .586 | .069 | .000 | 1.797 | 1.569 | 2.058 |
|  | 10001 to 20000 cases | .482 | .065 | .000 | 1.620 | 1.427 | 1.839 |
|  | 20001 to 40000 cases | .268 | .065 | .000 | 1.307 | 1.152 | 1.484 |
|  | 0 to 20 years | 1.237 | .044 | .000 | 3.444 | 3.162 | 3.751 |
|  | 20 to 40 years | .849 | .040 | .000 | 2.337 | 2.162 | 2.526 |
|  | 40 to 60 years | .460 | .039 | .000 | 1.584 | 1.469 | 1.709 |
|  | underweight | -.060 | .096 | .534 | .942 | .780 | 1.137 |
|  | normal_weight | -.127 | .034 | .000 | .881 | .824 | .942 |
|  | preobesity | -.071 | .033 | .030 | .931 | .873 | .993 |
|  | never_smoking | -.103 | .043 | .017 | .902 | .828 | .982 |
|  | quit | -.130 | .048 | .007 | .878 | .799 | .965 |
|  | vape | -.070 | .061 | .248 | .932 | .827 | 1.050 |
|  | never_drinking | -.168 | .035 | .000 | .845 | .789 | .906 |
|  | none_14days | -.008 | .034 | .806 | .992 | .928 | 1.060 |
|  | never_drugs | .201 | .035 | .000 | 1.223 | 1.141 | 1.311 |
|  | none_28days | .159 | .037 | .000 | 1.172 | 1.089 | 1.262 |
|  | have_diseases | -.170 | .029 | .000 | .844 | .798 | .892 |
|  | Constant | -.311 | .085 | .000 | .733 |  |  |
